# Codon-Driven Translational Efficiency Is Stable across Diverse Mammalian Cell States

**DOI:** 10.1371/journal.pgen.1006024

**Published:** 2016-05-11

**Authors:** Konrad L. M. Rudolph, Bianca M. Schmitt, Diego Villar, Robert J. White, John C. Marioni, Claudia Kutter, Duncan T. Odom

**Affiliations:** 1 European Molecular Biology Laboratory, European Bioinformatics Institute, Cambridge, United Kingdom; 2 University of Cambridge, Cancer Research UK Cambridge Institute, Cambridge, United Kingdom; 3 University of York, Department of Biology, York, United Kingdom; 4 Wellcome Trust Sanger Institute, Cambridge, United Kingdom; 5 Science for Life Laboratory, Karolinska Institute, Department of Microbiology, Tumor and Cell Biology, Stockholm, Sweden; CNRS - Université Montpellier 2, FRANCE

## Abstract

Whether codon usage fine-tunes mRNA translation in mammals remains controversial, with recent papers suggesting that production of proteins in specific Gene Ontological (GO) pathways can be regulated by actively modifying the codon and anticodon pools in different cellular conditions. In this work, we compared the sequence content of genes in specific GO categories with the exonic genome background. Although a substantial fraction of variability in codon usage could be explained by random sampling, almost half of GO sets showed more variability in codon usage than expected by chance. Nevertheless, by quantifying translational efficiency in healthy and cancerous tissues in human and mouse, we demonstrated that a given tRNA pool can equally well translate many different sets of mRNAs, irrespective of their cell-type specificity. This disconnect between variations in codon usage and the stability of translational efficiency is best explained by differences in GC content between gene sets. GC variation across the mammalian genome is most likely a result of the interplay between genome repair and gene duplication mechanisms, rather than selective pressures caused by codon-driven translational rates. Consequently, codon usage differences in mammalian transcriptomes are most easily explained by well-understood mutational biases acting on the underlying genome.

## Author Summary

Do mammalian cells employ codon usage alterations in groups of transcripts to control protein translation? Although long documented in prokaryotes, single-cell eukaryotes and protozoa, no convincing evidence has been presented to indicate this is the case in mammals. Nevertheless, many recent studies have asserted that codon usage can deviate from the genomic or transcriptomic background in a functionally impactful manner. For instance, one recent study suggests that mammalian genes associated with specific GO terms show ‘codon adaptation’ towards proliferation and differentiation. We have tested a number of closely related hypotheses by collecting and analyzing a comprehensive, genome-wide set of data that quantifies codons in mRNA as well as anticodons in tRNA transcriptomes in extreme cell conditions in human and mouse. The hypotheses our data test include suggestions that codon usage biases in mammals can be actively regulated to match: (a) most-highly differentially expressed protein coding genes, (b) condition specific GO gene sets, (c) housekeeping genes, (d) genes encoding ribosomal proteins, (e) proliferation-associated protein-coding genes. We found that of the codon usage signal that remains after accounting for gene set sizes and other genomic factors is caused largely by the underlying GC content of the genomes. In other words, codon usage biases postulated to exist within mammalian transcriptomes might be explained by small gene sets or via differences in GC content across the genome.

## Introduction

The degeneracy of the genetic code means that often several synonymous codons encode the same amino acid. Under the neutral theory of evolution, these synonymous codons should not be affected by selection, as they do not alter the amino acid sequence. However, genome-wide and gene-specific studies have revealed the existence of non-uniform codon usage, frequently referred to as codon bias, across all domains of life [1–3]. Codon bias affects a number of cellular processes, such as translational efficiency, peptide elongation and protein folding as well as exonic transcription factor binding, mRNA stability and splicing [4–12]. This suggests that using specific sets of synonymous codons at different times or in different cell types might have regulatory potential, meaning that natural selection could act upon the codon usage in expressed protein-coding genes. Indeed, prokaryotes and single-cell eukaryotes can actively regulate protein expression levels by adjusting codon usage and tRNA anticodon abundance [6, 7, 13, 14].

Whether codon usage is used to fine-tune levels of protein translation in mammals is actively debated (reviewed by [3, 15]). Mammals have a number of important differences when compared with single cell organisms, including variable genomic GC content, small effective population sizes, an expanded regulatory landscape, and multiple cell types, all of which have been speculated to underpin differences in what mechanisms can control transcription and translation in different organisms [2, 3, 16]. For instance, previous work [17–19] has shown that analysis of intergenic sequences flanking exons across evolution can predict genome-wide codon bias, and concluded that codon usage in complex genomes is predominantly determined by DNA mutations and only secondarily by selective forces acting on translated sequences.

Because codons are translated into amino acids by tRNAs, the rate at which a protein can be synthesized from an mRNA is influenced by the relationship between tRNA anticodon abundance and codon frequency within the mRNA. Any computational model of this relationship makes assumptions that reflect the underlying structure of the genome. For example, in all organisms, orphan codons that lack a corresponding tRNA are decoded using non-canonical pairing between codons and anticodons, commonly referred to as wobble base pairing [20]. Previous approaches to model wobble pairing have mainly focused on unicellular organisms and established decoding efficiency by assuming that highly expressed genes are more efficiently translated [1, 21]. Yet the extent to which each codon is translated by a specific tRNA via wobble base pairing differs between species [22, 23]. Consequently, these models cannot be readily applied to mammals. Additionally, the genomes of multicellular organisms generate hundreds of different cell-types, all of which are characterized by distinct sets of highly expressed genes [24]. However, widely used methods, such as the tRNA Adaptation Index (tAI) [1], have optimized their wobble base pairing models in more simple single-cell organisms using only a single high-expression transcriptome [9].

Furthermore, the multi-cellularity of mammals also means that the frequency of a codon across the whole transcriptome may vary between cell types. Similarly, cell-type-specific tRNA expression can generate appreciably different pools of expressed tRNA anticodons [25, 26]. However, prior approaches for estimating translational efficiency in single cell organisms do not account for variability in tissue-specific mRNA expression (Codon Adaptation Index) [27], while other methods, such as tRNA adaptation index (tAI) approximate tRNA abundance using gene copy number [1].

We previously evaluated the relationship between codon usage and anticodon abundance during different stages of mouse development [28]. The transcriptomes of both mRNAs and tRNAs were highly variable. However, comparing the expression levels of each codon (summed across all transcribed mRNAs) with the expression of the corresponding anticodon (summed across all relevant transcribed tRNAs) revealed high stability throughout mouse development. Other studies using ribosome profiling have revealed consistent rates of translation genome wide, independent of tAI or mRNA transcript levels [29].

By contrast, other recent work has suggested that functional differences do exist in codon usage in subsets of mammalian protein-coding genes—be it highly expressed genes [30, 31], tissue specific genes [32], housekeeping genes [33] or genes associated with different GO terms [34]. These studies hypothesized that the changes in codon usage observed between specific subsets of the transcriptome are coupled to differences in tRNA anticodon abundances, suggesting a mechanism whereby translational efficiency is differentially regulated between gene sets.

To resolve whether codon usage influences translation in mammalian cells, we designed experiments to quantify the codons used within cell-type specific mRNA transcriptomes, as well as the anticodons present in the corresponding pool of expressed tRNAs. Because recent publications have suggested that codon usage differs most between cells that are undergoing proliferation or differentiation [34], we chose to compare the codons and anticodons used in (i) rapidly growing cancer cells *in vitro* and in (ii) highly differentiated, quiescent hepatocytes *in vivo*. If codon usage affects translational efficiency in mammalian cells, we reasoned that a proliferating cancer line and resting differentiated cells should display divergent tRNA abundance and concomitant codon bias.

While we had previously only investigated codon usage between whole transcriptomes [28], here we perform new experiments and analyses designed to evaluate codon usage differences amongst distinct sets of genes. Our results do not seem to support a model wherein codon usage is functionally maintained to match with tRNA anticodons in order to control translational efficiency in mammalian cells. Our conclusions are consistent whether considering highly expressed genes, differentially expressed genes, Gene Ontology-specific categories of genes, or the entire transcriptome. Instead, the tRNA pool in mammals appears to be equally efficient at translating any transcriptome, regardless of cell type or condition. Our results support prior studies that have suggested that differences in codon usage between mammalian gene sets are most likely driven by underlying sequence features such as GC content [19, 35].

## Results

### 1. Deviation of codon usage from exonic background is partially explained by random sampling

In mammals, the codon usage across the entire transcriptome is highly stable, whether considered across species, diverse tissues or during development [26, 28]. However, it has been postulated that distinct subsets of genes utilize specific codons to modulate translation rates based on the availability of a specific anticodon tRNA pool [32–34]. Indeed, a recent report [34] indicated that proliferation and differentiation genes use distinct sets of codons, and further suggested that these codon frequencies have been evolutionarily selected to afford cell-type specific translational (and thus functional) optimization. We asked whether these distinct codon deployments (that is, observed codon biases) could be explained in part by the small number of genes within such gene sets, potentially leading to mis-interpretation of stochastic variation as having functional relevance.

**Fig 1:**
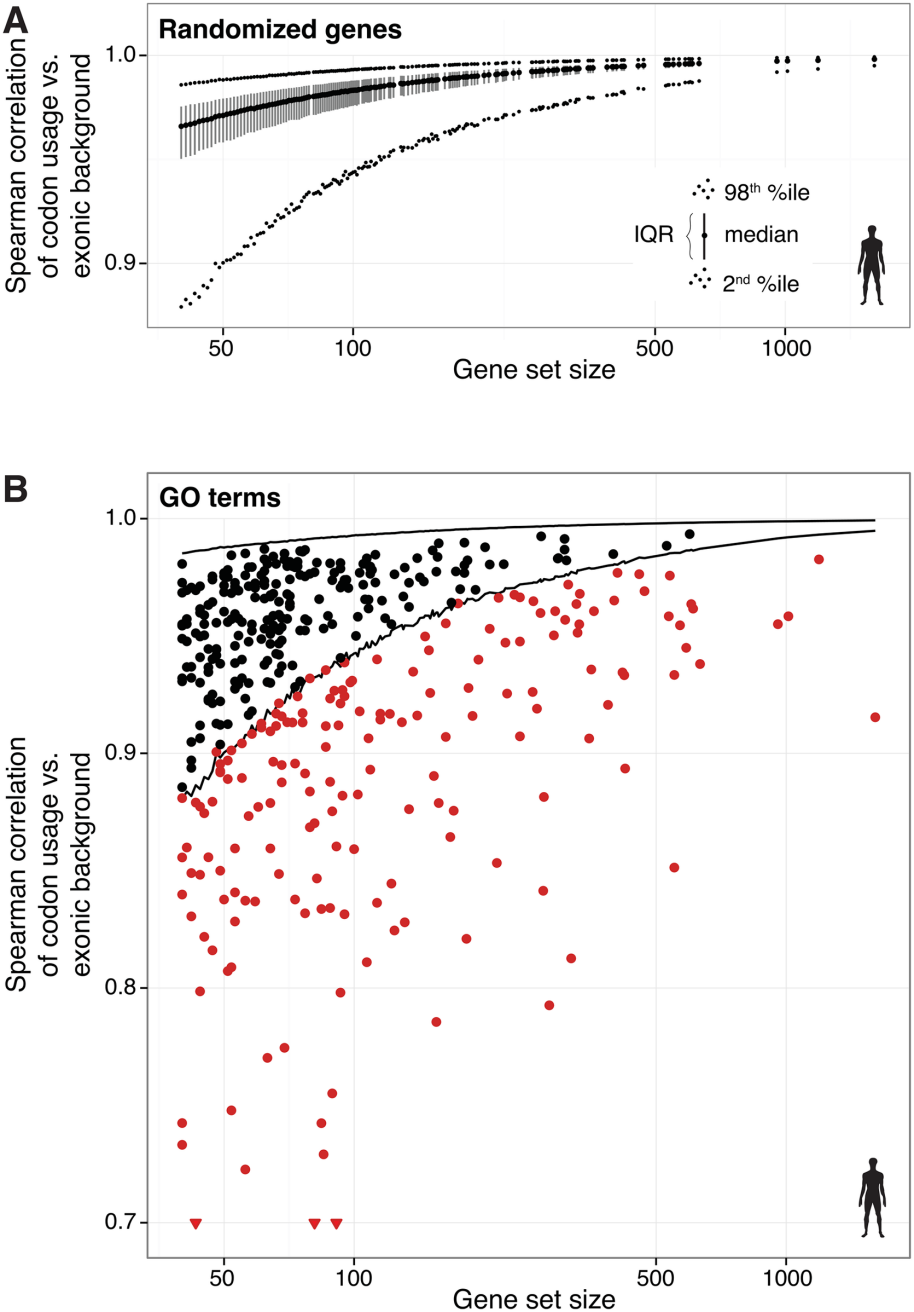
Gene set sample size correlates with codon usage variability. (**A**) Genes were randomly sampled from the human genome to create sets of different sizes corresponding to GO term set sizes (x-axis). The codon usage of each set was calculated and correlated with the exonic background (Spearman’s rank correlation coefficients (ρ); y-axis). For each set size, the distribution of 10 000 repeated samples is summarized in the figure by a vertical segment connecting the 1^st^ with the 3^rd^ quartile (interquartile range, IQR); the median is indicated as a black dot; the 2^nd^ and 98^th^ percentile are indicated with smaller dots. (**B**) For each human GO gene set, the Spearman correlation between its codon usage and the codon usage of the exonic background is plotted (y-axis) against the number of genes in this set (x-axis). Each point in the graph corresponds to a GO term. For all gene set sizes, the distributions from (A) were used as a background to empirically test the null hypothesis that the codon usage correlation of a GO term is explained by random sampling alone. GO terms whose codon usage significantly diverges from the background (FDR-adjusted *p* < 0.05) are colored in red; GO terms without significant divergence are colored in black. The lines indicate the 2^nd^ and 98^th^ percentile of the random distribution from (A).

First, we estimated the stochastic variation in codon usage generated by sampling sets of genes randomly from the human genome; set sizes were chosen to match the number of genes associated with actual GO terms that range from 40 to 1 611 genes (S1 Table). For each set size, we repeated this sampling method 10 000 times. For all sets of genes, we then calculated the Spearman correlation between their codon usage and the codon usage of the entire human genome (exonic background) (Methods; Fig 1A). Our analysis confirmed that smaller gene set sizes have substantially greater variation in codon usage than larger sets. Overall, this analysis reveals the variation in codon usage expected purely by chance in gene sets with equal gene numbers to human GO terms.

Second, to evaluate whether differential codon usage of genes associated with different GO terms can be explained by stochastic variation, we overlaid their codon usages upon this randomly generated distribution (Fig 1B). For each individual gene set associated with a GO term, we computed empirical *p*-values to test whether the observed correlation differed from all genes. More than half (233 out of 410, 57%) of the GO terms fell within the range expected by chance (Fig 1B, black dots). Therefore, any codon usage variations observed in genes associated with these GO terms are indistinguishable from random.

However, for the remaining 43% of GO terms (177 out of 410), the observed correlations were smaller than expected by chance and cannot be explained by random sampling (Fig 1B, red dots, empirical *p*-value < 0.05). Nevertheless, the existence of a substantial codon usage bias in many GO categories led us to design experiments to identify the underlying molecular mechanisms, as well as to test whether codon usage differences have functional implications for translation.

### 2. Codon adaptation to tRNA anticodon pools is not cell-type specific

We then asked whether codon usage has been optimized for translation: (i) across the entire transcriptome for a specific tissue [28], (ii) for highly expressed, tissue-specific genes [30–32], (iii) for GO categories [34], and (iv) for house-keeping [33], ribosomal [36] and proliferation-driving genes [37].

**Fig 2:**
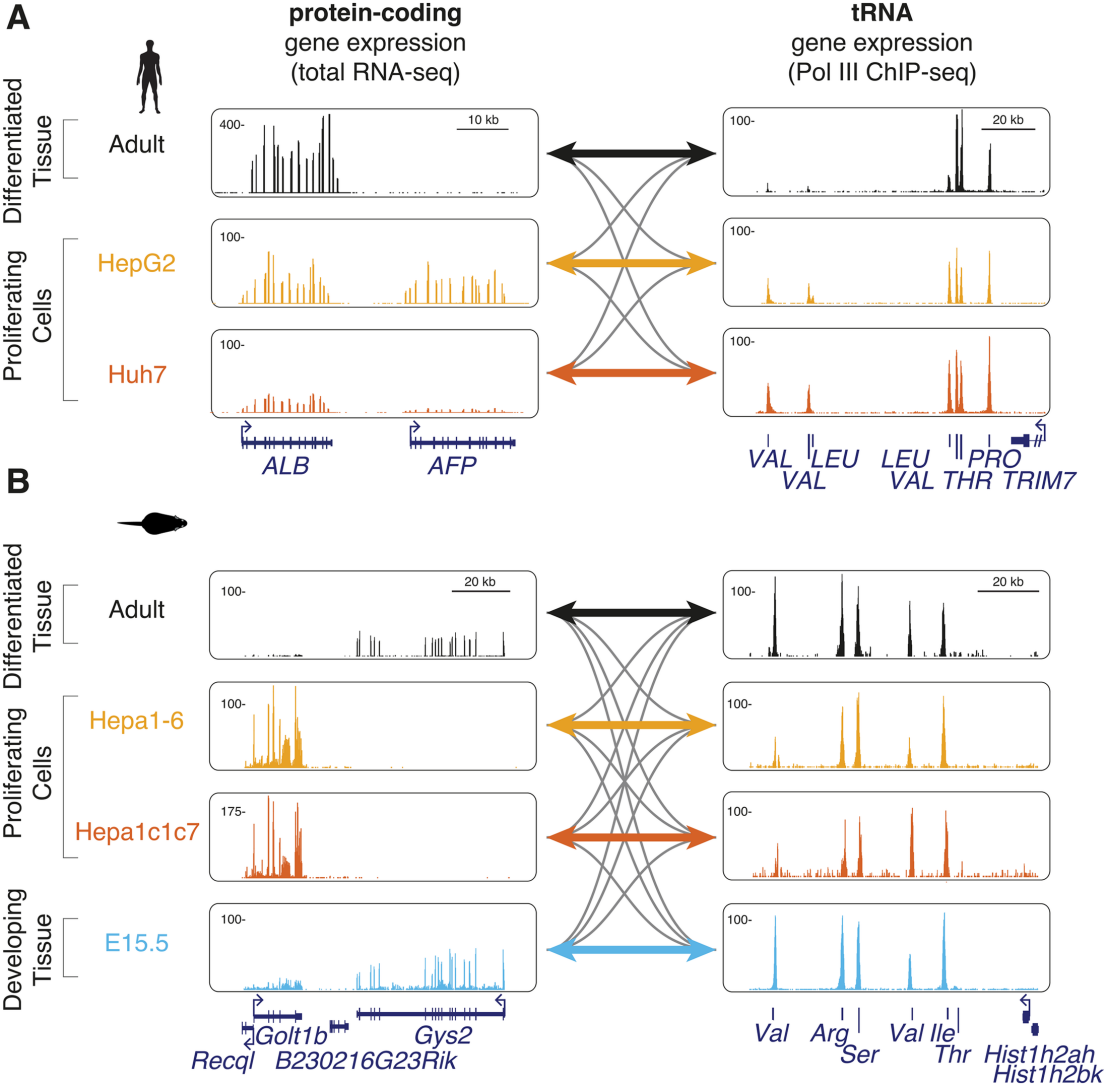
Cell type-specific expression of protein-coding and tRNA genes. Protein-coding and tRNA gene expression levels in (**A**) human and (**B**) mouse were measured in proliferating hepatic cells (HepG2 and Huh7 in human, Hepa1-6 and Hepa1c1c7 in mouse; yellow and red tracks), quiescent adult (black tracks) and developing embryonic E15.5 (blue tracks) liver tissue. Shown are examples of differentially regulated protein-coding (left) and tRNA (right) genes. Colored and grey bidirectional arrows represent the matching and mismatching codon–anticodon conditions compared, respectively. The y-axis of each track specifies normalized read density. Scale bars show length of genomic regions in kilobases (kb).

To explore the extent of codon adaptation, we generated data from mammalian primary tissues and cell lines with divergent cellular phenotypes. We reasoned that, if the codon usage of sets of genes that drive proliferation have been optimized, then direct comparison of codon usage in quiescent tissues and their derived cancer cell lines should be a simple and powerful model system to test this hypothesis. Therefore, we chose adult liver as a fully differentiated and highly homogeneous organ and two separate, highly proliferating liver cancer cell lines, in both mouse (Hepa1-6 and Hepa1c1c7) and human (HepG2 and Huh7) (Fig 2). In mouse, we additionally used embryonic day 15.5 developing liver as an actively differentiating tissue.

Translational efficiency can be estimated by comparing codon usage in the mRNA transcriptome with tRNA anticodon abundance [8, 28]. Therefore, in each tissue we quantified protein-coding gene expression levels using strand-specific, total RNA-sequencing, as well as matched tRNA transcriptomes using Polymerase III (Pol III) chromatin immunoprecipitation followed by sequencing (ChIP-seq) [26, 28, 38–43] (S1 Fig, Methods). Between two and four biological replicates were generated for each experiment (S2–S5 Figs, S2–S5 Tables).

Principal components analysis (PCA) using gene expression counts revealed that our samples cluster by cell type (S6 Fig). Using our transcriptome-wide sequencing data, we observed that 72% (14 283/19 850) of annotated protein-coding and 69% (362/523) of tRNA genes were expressed in at least one sample, with 72% (10 295/14 283) of the protein-coding and 93% (338/362) of the tRNA genes expressed in one sample are also active in one or more other human cell types (S6 Fig, S2–S5 Tables). On average, 40% of all protein-coding and 29% of all tRNA genes were differentially expressed between any pair of human cell types (DESeq2 with FDR cutoff 0.01; S6 and S7 Figs, S6–S9 Tables). A similar trend was observed for mouse, consistent with previous studies [26, 28].

**Fig 3:**
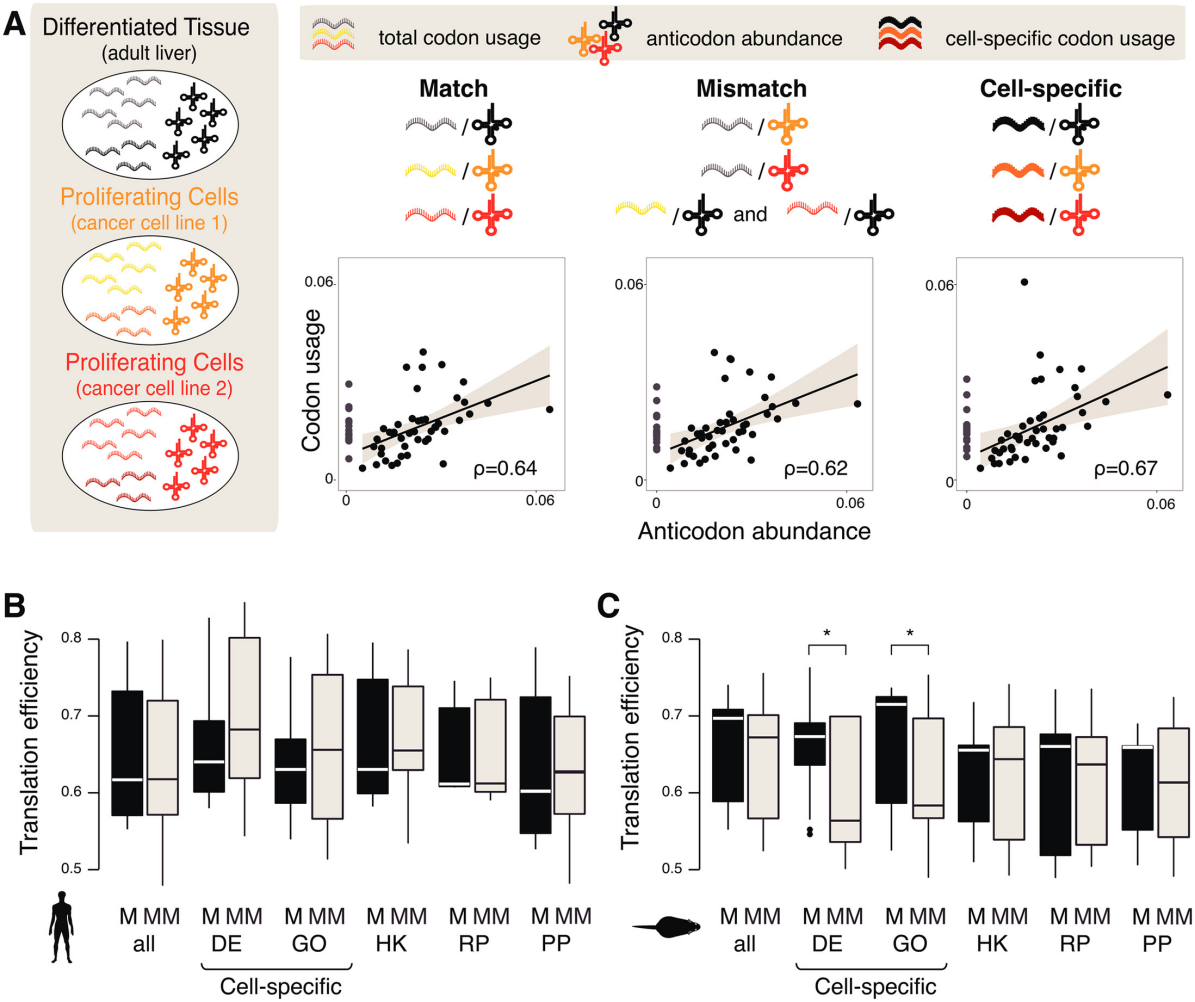
Correlation of codon usage and tRNA abundance shows no consistent evidence of translational adaptation. (**A**) Schematic representation of the codon–anticodon relationship for each color-coded cell type. Comparisons were performed for “Match (M)”: between global codon and anticodon abundances from the same cell type; “Mismatch (MM)”: between codon abundance from one cell type and anticodon abundance from a different cell type; and “Cell-specific”: between codon abundance for a subset of genes and anticodon abundance for all conditions (encompassing both match and mismatch). Examples for each comparison are shown by plotting proportional frequencies of Pol III binding to tRNA anticodons (x-axis) and transcriptomic codon frequencies weighted by expression levels (y-axis) obtained from RNA-seq data. Spearman’s rank correlation coefficients (ρ) are indicated. Anticodons for which there are no corresponding tRNA genes (gray dots) were excluded when calculating the correlation coefficients. The gray area within plots represents the 95% confidence interval. Boxplots show the resulting codon–anticodon correlations for all pairwise comparisons for (**B**) human and (**C**) mouse. “Match”, “Mismatch” and “Cell-specific” samples were correlated as in (**A**). Translational efficiencies are shown as correlations of the codon pool for all genes (“all”), the 200 most highly differentially expressed (“DE”) protein-coding genes, condition-specific gene ontology (“GO”) term gene sets, house-keeping (“HK”), ribosomal (“RP”) or proliferation-driving (“PP”) protein encoding genes with either the anticodon pool of the same condition (“M”) or any other condition (“MM”). Asterisks above the bars indicate significant differences (one-tailed Mann–Whitney–Wilcoxon test; significance codes Bonferroni-corrected *p*-values: 0–0.001***, 0.001–0.01**, and 0.01–0.05*) for a given contrast.

If codon usage is optimized for tissue-specific gene expression, then the mRNA codon usage and tRNA anticodon abundance should correspond better within a given cell type than between different cell types (Fig 2). For example, one might expect the mRNA molecules involved in proliferation to be more efficiently translated by the tRNA anticodon pool in cancer cells than by the tRNA anticodon pool present in terminally differentiated cells. Using the expression levels for protein-coding and tRNA genes, we derived the abundances of all 61 amino acid encoding triplet codons and 45 tRNA anticodons in human (and 46 in mouse) for each cell type (Fig 3A).

We next calculated the codon–anticodon correlation of matching mRNA and tRNA transcriptomes in each cell type (hereafter named “matching” conditions) as a proxy for global translational efficiency. We also correlated the mRNA transcriptome of each cell type with the tRNA transcriptome of other cell types (hereafter named “mismatching” conditions) (Fig 3B). If selection for translational efficiency occurs, the correlation between the “mismatch” conditions should be lower than the correlation between the “match” conditions.

We observed that the codon–anticodon correlations computed across global transcriptomes were not significantly different between the match and the mismatch conditions (one-tailed Mann–Whitney–Wilcoxon test, Bonferroni-corrected *p* = 0.38; Fig 3B). Our results indicate that the mRNA transcriptome of one cell type is translated with similar efficiency by the tRNA transcriptome of any other cell type. More formally, there is no evidence to reject the null hypothesis that the tissue-specific transcriptome of each cell type we measured can be equally well translated by the tRNA transcriptome of any other cell type.

If translational efficiency is optimized for tissue-specific genes, then their codon usage in each cell type should be best matched to the same cell type’s tRNA anticodon abundance (Fig 2). We identified the 200 most significantly differentially expressed mRNA genes between liver and the derived cancer cell lines (S7 Fig, S10 and S11 Tables) and compared the translational efficiencies of these gene sets using matching and mismatching sets of tRNA anticodons (Methods). Our analysis of human samples showed no significant differences between the translational efficiencies of proliferating and quiescent liver cells (one-tailed Mann–Whitney–Wilcoxon test, Bonferroni-corrected *p* = 0.83) (Fig 3B). Our results were consistent with a prior report showing that there seems to be no selection pressure on codon usage to optimize translational efficiency in human tissues [35].

Additionally, we identified the GO terms that most distinguished healthy and cancerous liver samples (Methods) and performed a similar analysis on the component genes (Fig 3B, S10–S13 Tables). In human, we observed no difference in the codon–anticodon correlations between the identified GO categories and the whole transcriptome (one-tailed Mann–Whitney–Wilcoxon test, Bonferroni-corrected *p* = 0.72). We also inspected selected sets of genes in which optimized translational efficiencies could be expected either due to their basic cellular functions or due to their roles in proliferation. Similarly to the comparisons described earlier, no significant differences were observed between the matching and mismatching conditions for housekeeping, ribosomal or proliferation-driving genes in human (one-tailed Mann–Whitney–Wilcoxon test, Bonferroni-corrected *p* = 0.62, *p* = 0.23 and *p* = 0.53, respectively, Fig 3B, S10 and S11 Tables).

We avoided making assumptions about the underlying non-canonical base pairing efficiency [28], but note that incorporating information on wobble-pairs or using alternative measures of translational efficiency such as the tAI do not alter our conclusions (see Methods and S9 and S10 Figs). Translational efficiency can be affected by the rate of translational initiation since the first codons in the mRNA sequence are generally translated with lower efficiencies [7, 44]. We excluded the possibility that variation in transcription initiation strongly affected our conclusions by repeating the analyses above using the first ten codons of each transcript (see Methods S11 Fig, S12 and S13 Tables).

In sum, we performed experiments specifically designed to identify whether codon usage is optimized for either the entire or specific subsets of the mammalian transcriptome. Our results provide no evidence for optimization of translational efficiency by cell-type-specific codon usage in human tissues, even for subsets of highly tissue-specific transcripts (S7 Fig). For mouse, the results were slightly less conclusive: for tissue-specific genes and genes associated with GO terms we find evidence against the hypothesis of no difference in translational efficiency (Fig 3C). However, the difference in upregulated genes between match and mismatch can be explained by shifts in translational efficiency driven by the Hepa1-6 tRNA pool; for all other tRNA pools, no differences were observed (S8 Fig). Nevertheless, given our observations in Hepa1-6 cells, we used an independent alternative approach to further test the hypothesis of codon bias by comparing sets of genes within rather than across each condition.

### 3. Observed codon usage differences in GO sets are inconsistent with translational optimization

We directly tested the hypothesis that the codons within cell-type specific gene sets of GO terms are optimized for tissue-specific translation, which has been suggested as an adaptive process [34]. In other words, we asked whether the cell-type specific genes associated with GO terms identified in the prior section were optimized for translational efficiency in their corresponding cell type relative to all remaining GO categories.

**Fig 4:**
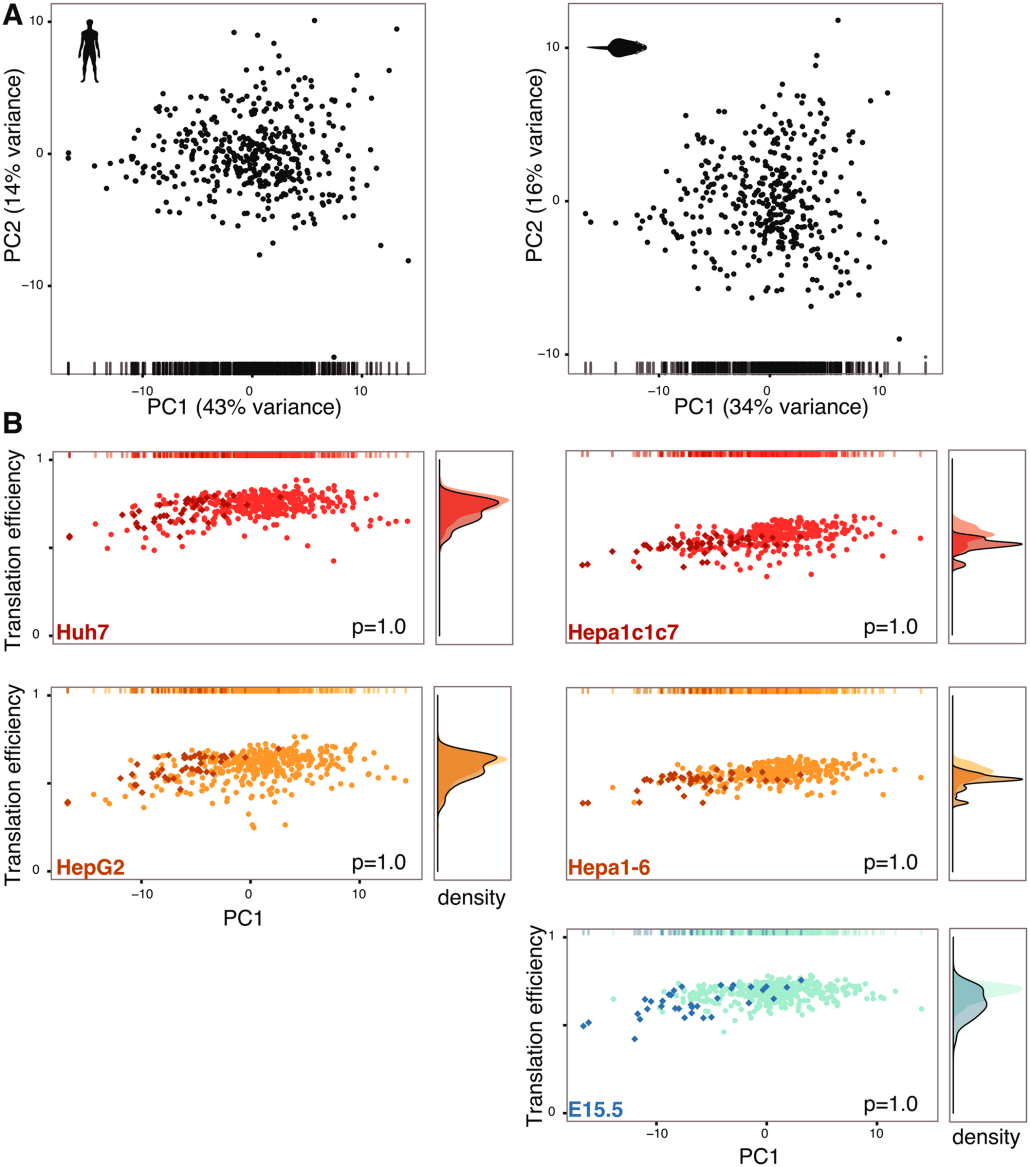
Translational efficiency does not correlate with cell-type-specific codon usage. (**A**) Factorial map of the principal components (PC) analysis of genomic codon usage per Gene Ontology (GO) term in adult liver (left: human; right: mouse). Each point corresponds to one GO term. The position of each point is given by the first (x-axis) and second (y-axis) principal component of the corresponding GO term’s codon usage. The proportion of variance explained by each PC is shown in parentheses. The bar at the bottom corresponds to the x-coordinate of each GO term. (**B**) Cell type-specific translational efficiency of each GO term is plotted against their first principal component shown in (**A**) (indicated by bar at top; left: human cell types; right: mouse cell types). The x-axes in (**A**) and (**B**) are identical. The y-axis shows the cell type-specific mean translational efficiency of each GO term. Darker colors and diamond symbols correspond to enriched GO terms (using gene set analysis of differentially expressed genes, empirical *p* < 0.001). Enrichment of cell lines and E15.5 is defined by contrast with adult liver. Lighter colors are non-enriched GO terms. The panel on the right hand side of each panel shows the density of the distributions of translational efficiencies for enriched (solid trace line) and non-enriched (no trace line) GO terms. The numbers at the bottom right of each plot correspond to the Bonferroni-corrected *p*-value of a one-sided Mann–Whitney–Wilcoxon test of the null hypothesis that no difference exists between the translational efficiency of enriched and non-enriched GO terms.

First, we computed the codon frequency of the longest transcript for each gene associated with each GO term (Methods). Similar to Gingold et al. [34], we observed differences in codon usage in genes associated with different GO categories (Fig 4A, S12 Fig). For example, GO terms that are specific to human liver cancer cell lines have negative values on PC1 (Fig 4B), while GO terms enriched in fully differentiated human liver consistently have positive values. However, since this analysis does not account for differences in gene expression between cell types, codon frequency differences may not correspond to differences in translational efficiency across the actual transcriptome.

**Fig 5:**
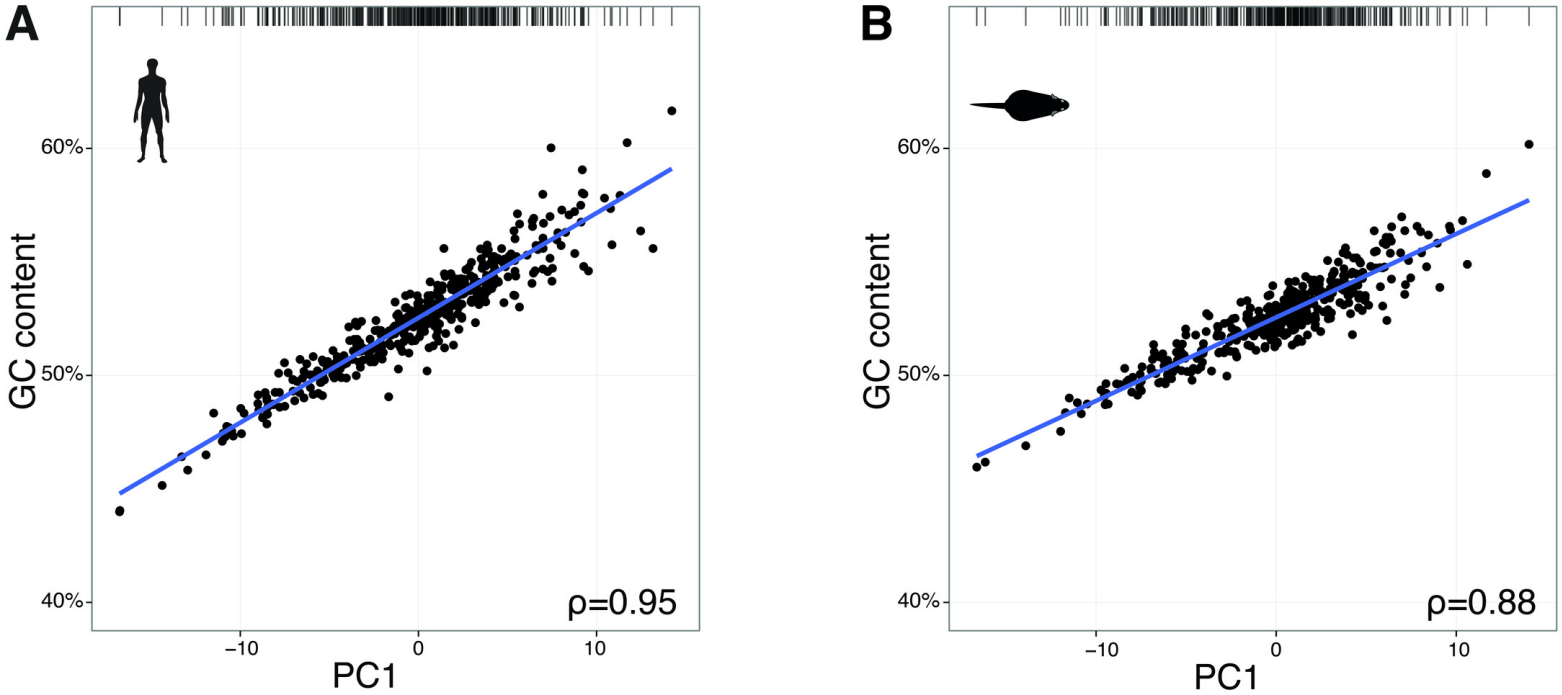
Translational efficiency correlates with transcriptomic GC content. The first principal component (PC) of genomic codon usage per Gene Ontology (GO) term (Fig 4A) of adult (**A**) human and (**B**) mouse liver (indicated by bar at the top) is plotted (x-axis) against the GC content for each gene set. Spearman’s rank correlation coefficients (ρ) are shown.

For each cell type, we therefore computed the translational efficiency of each GO term gene set using its codon frequencies weighted by gene expression and the corresponding tRNA anticodon abundance pool. For each cell type in Fig 4B, the density curve on the right hand side of each panel reveals that the GO terms identified as enriched in each cell type are not optimized for translation relative to the remaining GO categories. Since translational efficiency of genes does not correlate with variability in GO term codon usage, we sought to investigate other genomic features that may explain the observed variation. Prior studies have suggested that the local density of GC base pairs has a substantial influence upon the evolution of codon usage across the genome [19, 35]. Indeed, we confirmed that PC1 in Fig 4A is strongly correlated with GC content across genes associated with GO terms for both human (Spearman’s ρ = 0.95) and mouse (Spearman’s ρ = 0.88) (Fig 5).

In sum, our results indicate that differences in codon usage of genes associated with GO terms are not associated with differences in translational efficiency, but are instead most consistent with a simple model wherein the genomic GC content shapes codon usage in transcriptomes.

## Discussion

Understanding the regulatory mechanisms controlling how DNA is converted to RNA, which is then translated into protein sequences, is a key challenge in molecular biology. In prokaryotes and unicellular eukaryotes, protein levels can be regulated by complementary changes in codon and anticodon abundance [6, 7, 14]. Extensive theoretical and experimental research has explored how pressures from the large population sizes and rapid growth have helped to shape the prokaryotic genome features that enable codon bias [1, 3, 19, 45]. These studies have concluded that natural selection upon codon usage can act more efficiently in small genomes like *E*. *coli* where there are fewer anticodons represented in the tRNA pool, and where the number of tRNA gene copies per anticodon family is modest [1]. Practically, these features mean that in prokaryotes, any changes in tRNA gene expression directly impact translational efficiency, unless compensation takes place in the codon pool [45].

Our experimental and computational analyses challenge recent reports that codon usage is also selectively shaped to fine-tune translation in mammals [30–34, 36]. First, our analysis demonstrates the existence of high stochastic variability in codon usage when small sets of genes are analyzed. Indeed, over half of the GO sets showed codon usage differences that were indistinguishable from that expected by chance. However, this does not preclude the possibility that a subset of genes in specific cell-types have adapted codon usage to optimize translational efficiency, as suggested by a recent study [34]. To explicitly test this, we contrasted the rates of translational efficiency between quiescent (healthy) cells and highly-proliferating cancer cells in both human and mouse.

Our approach to measure translational efficiency by comparing codon and anticodon pools has two limitations. First, our measures of tRNA gene expression relied on Pol III binding, which captures tRNA gene utilization at every locus and cannot account for post-transcriptional tRNA processing, amino acid charging, or covalent base modifications, all of which can affect the rate and fidelity of translation [46–49]. Second, our measures of mRNA transcription cannot account for post-transcriptional folding and stability [50, 51]. Finally, although liver consists of over 70% hepatocytes and is highly homogeneous, there exist minor cell types that may contribute modestly to our mRNA and tRNA transcriptomes. Importantly, however, all adult cell types in the healthy liver tissues we used are in a quiescent and nonproliferating state, thus serving as an excellent comparitor to highly proliferative cancer cells.

Despite an overall lack of evidence for optimized translational efficiency between cell types, differences in codon frequencies between sets of genes with specific functions do exist. Even in these small sets of genes, differences in codon frequencies are not correlated with differences in translational efficiency. In other words, genes associated with cell-type specific GO terms are not better optimized for translational efficiency than are any other set of genes. Instead, our data suggest that these differences are driven by underlying GC content, a common confounding factor in many sequence analyses of codon bias and genome evolution [19, 52]. Mammalian genomes exhibit variation in GC content over larger genomic regions, the cause of which is still not fully understood. Differences in GC content within a genome have been linked to differences in mutational processes across the genome, such as biased gene conversion [53–55] and/or paralogous gene expansion [56]. Thus, the difference in GC content between gene sets might be the result of evolutionary forces unrelated to codon usage.

Ribosome profiling studies in mammals have shown that synonymous codons with different tAI are generally translated at a similar speed, and initiation is the rate-limiting step of protein synthesis. Accordingly, molecular elements (such as translation initiation factors) or genomic features at the initiation site of the transcript control the overall rate of translation, rather than mRNA codon composition [29, 57]. By contrast, other studies concluded that elongation is slowed at wobble positions [58]. These opposing results cast doubt on the overall role of codon usage in the control of translation.

Regardless, in mammals, the available evidence suggests that mutational bias is a sufficient explanation for variations in codon usage between sets of genes. Compared to prokaryotes, the mammalian genome is better optimized for complex transcriptional and post-transcriptional regulation than for codon adaptation. Mammals have complex and multi-layered regulatory machineries and, in particular, widespread use of distal regulatory elements and epigenetic modifications. Our results argue strongly that prokaryotes and mammals differ in the extent to which translational efficiency and codon biases are relied upon as a regulatory mechanism.

## Methods

### Ethics statement

The investigation was approved by the Animal Welfare and Ethics Review Board and followed the Cambridge Institute guidelines for the use of animals in experimental studies under Home Office license PPL 70/7535. Human liver samples were obtained under Human Tissue Act license 08-H0308-117 from the Addenbrooke’s Hospital at the University of Cambridge with patients’ consent.

### Experimental description

#### Tissue preparation

Liver hepatocellular carcinoma cell lines HepG2 and Huh7 from human and Hepa1-6 and Hepa1c1c7 from mouse were grown to 80% confluency in DMEM (Sigma) supplemented with 10% fetal bovine serum (FBS) and antibiotics (100 μg/μl penicillin and 100 μg/ml streptomycin) at 37°C and 5% CO_2_. At least two independent cell passages or biological samples were obtained for each cell line. The HepG2 and Huh7 cell lines were genotyped by short-tandem repeat genetic profiling (STR) using the PowerPlex_16HS_Cell Line panel and analyzed using Applied Biosystems Gene Mapper ID v3.2.1 software by the external provider Genetica DNA Laboratories (LabCorp Specialty Testing Group). Healthy mouse liver were isolated from *Mus musculus domesticus* C57BL/6 (two and four males for ChIP- and RNA-seq, respectively, 10 to 12 weeks old, obtained from Charles River). We used healthy adult human livers (two males for ChIP-seq and three males for RNA-seq). Cells and liver tissue were either post-mortem cross-linked or fresh-frozen in liquid nitrogen.

#### Chromatin immunoprecipitation followed by high-throughput sequencing (ChIP-seq) library preparation

Pol III ChIP-seq assays were performed as previously described [26]. Briefly, cells were fixed in 1% formaldehyde (v/v), lysed, sonicated and then incubated with antibodies recognizing antigen POLR3A, the RPC1/155 subunit of Pol III. Immunoprecipitated DNA was end-repaired, A-tailed, ligated to Illumina sequencing adapters, amplified by 18 cycles of PCR and size selected (200–300 bp). DNA fragments were 36 bp single-end reads sequenced on an Illumina Genome Analyser IIx or HiSeq2000 according to manufacturer’s instructions (detailed under ArrayExpress submission).

#### Total RNA-sequencing (RNA-seq) library preparation

Total RNA was extracted using Qiazol reagent (Qiagen) from fresh-frozen cells and liver tissue and treated with DNase (Turbo DNase, Ambion). RNA samples were ribosomal RNA depleted (RiboZero, Epicenter). Strand-specific libraries were prepared using d/UTPs [59] and multiplexed (Illumina TruSeq kit), and 75 bp paired-end sequenced on an Illumina HiSeq2000 according to manufacturer’s instructions.

### Computational analysis

#### Canonical coding sequences and codon usage

Each gene’s codon usage was determined from its canonical coding sequence. For each gene, the canonical coding sequence was defined as the longest annotated, valid coding sequence. Coding sequences were downloaded from Ensembl [60]. We used the human reference genome GRCh38 (GenBank 2339568) and mouse reference genome GRCm38 (GenBank 1700338), respectively. We called a coding sequence “valid” if it starts with the start codon ATG, ends with a stop codon (TAG, TAA or TGA) and its length is divisible by 3. Genes where no valid coding sequence was annotated were excluded from the analysis. Codon usage was then determined by counting the frequency of each codon in the canonical coding sequence.

#### Sample size effect

To assess whether gene set size has an influence on the variation in codon usage, we sampled random sets of genes and calculated the correlation between their codon usage and the genomic background. We used the Gene Ontology Association file for *H*. *sapiens* available from http://www.geneontology.org/gene-associations/submission/ (version 1.224, submission date 2012-02-20) to map GO terms to their gene sets. We excluded mitochondrial genes, and genes for which we could not find valid coding sequences. We then created random gene sets from the set of all annotated protein-coding genes, with sizes equal to the cardinality of actual GO term sets with at least 40 genes. For each set size, we created 10 000 random gene sets. For each sampled gene set, we calculated the aggregate codon usage as the sum of the genes’ codon frequencies. We then calculated the Spearman correlation between each sampled gene set’s codon usage and the genomic background codon usage, which we defined as the aggregate codon usage of all annotated canonical coding sequences.

Next, we calculated the Spearman correlation between the aggregated codon usage of each actual GO term gene set and the exonic background codon usage [28]. From this, we determined whether a given GO term’s codon usage diverged more significantly from the genomic background than expected by chance: We calculated an empirical *p*-value as the fraction of random gene sets of the same size whose codon usage correlation was less than that of the GO term. In other words: the fraction of random gene sets whose codon usage correlation was smaller. We called GO terms with FDR-adjusted *p* < 0.05 as having a codon usage that diverges more from the genomic background than expected by chance.

#### Quantification of RNA-seq data

RNA-seq mapping and quantification was performed using the iRAP pipeline [61]. RNA-seq libraries were mapped against the human reference genome GRCh38 (GenBank 2339568) and mouse reference genome GRCm38 (GenBank 1700338), as appropriate, using TopHat2 [62] with default parameters after quality filtering with a minimum Phred score of 10 and trimming of low-quality bases at the end. Read pairs were mapped with an insert size of 350 bp and a mate standard deviation of 100 bp. Gene-level expression quantification was performed using htseq2 [63] over protein-coding genes. For the subsequent analyses, only annotated protein-coding genes on the nuclear autosomes were used.

#### Quantification of ChIP-seq data

ChIP-seq libraries were mapped to the human reference genome GRCh38 and the mouse reference genome GRCm38, as appropriate, using BWA [64], writing a maximum of 50 alignments per read (parameter “-n 50”) after quality filtering using Reaper [65] with parameters “-nnn-check 3/5” (discard reads with 3 “N”s out of 5 bp), “-qqq-check 43/9” (discard reads with median Phred score less than 49 in a 9 bp-window) and trimming to a common length of 30 bp. Multi-mapping reads were then reallocated using the method from [26], before tRNA gene expression was quantified by counting reads falling onto the tRNA gene and within the flanking regions 100 bp up-and downstream, and taking their mean.

#### Filtering

All subsequent analyses were performed independently on the human and mouse samples. Only genes that were expressed in at least one of the assayed conditions were used. For mRNA, we required at least one library replicate to have a nonzero count for a gene to be included. For tRNA, a stricter filter was used to account for ChIP noise: first, library size factors were estimated using the method from Anders and Huber [63]. Next, to be considered expressed, we required that a gene had a normalized count of at least 10 in all replicates of at least one assayed condition. We also restricted our analysis to tRNA genes from nuclear autosomes.

#### Differential expression

For both mRNA and tRNA in human and mouse, differential expression analysis was performed using DESeq2 [66] with the default model for all pairwise combinations of tissues.

#### Gene set analysis

Gene set analysis was performed using Piano [67] using the same set of GO terms and associated genes as in the rest of the analysis. For mouse, the human Gene Ontology Annotation was translated by transforming human gene names to mouse gene names. We used the results from the differential gene expression analysis, and in particular the FDR-adjusted *p*-values of evidence for differential expression, as the test statistic for the gene set analysis. Furthermore, the log_2_ fold change was used to indicate the “direction” of enrichment. We then ran Piano with default parameters. We called GO terms significantly enriched if they had an adjusted *p*-value for directional enrichment (either “dist.dir.up” or “dist.dir.dn”) of *p* < 0.001.

#### Translation efficiency

To estimate translational efficiency of a gene set, *GS*, from a given mRNA transcriptome by a given tRNA pool, we calculated the gene set’s aggregate codon usage *CU* for codon *c* as follows:

$${CU}_{c}\;=\;\sum_{g\in GS}{CU}_{cg}\;\cdot \frac{x_{g}}{l_{g}}$$

That is, we sum the codon frequencies of the canonical coding sequences of each individual gene, multiplied by the estimated gene expression *x*_*g*_, normalized by the coding sequence length *l*_*g*_. We also summed the estimated tRNA gene expression of isoacceptor tRNAs to calculate anticodon frequencies. We then calculated the Spearman correlation between the codon usage and the given tRNA pool’s anticodon frequencies by pairing all codons to their corresponding anticodons. Wobble base pairings were thus ignored.

To confirm that this method yielded reliable results, we also calculated translational efficiency in two different ways and checked that our results remained consistent. First, we proceeded as above but accounted for unpaired codons (that is, codons without corresponding anticodons) by pairing them with their unique wobble-paired anticodon when calculating the correlation. Secondly, we calculated the tRNA adaptation index (tAI) [1] of each gene in the gene set individually, with two modifications: (i) The tGCN_*ij*_ (tRNA gene copy number) in the calculation of *W*_*i*_ was replaced by the tRNA abundance estimated from tRNA gene expression; (ii) We used the “initial” *s*-values, rather than optimised ones, because the optimization procedure by dos Reis [1] assumes that highly-expressed genes have more optimal codon usage, which would introduce circularity into our reasoning. After calculating the per-gene tAI, we computed a weighted average of the genes using their gene expression.

#### Translational efficiency comparison

To test whether an mRNA gene set could be more efficiently translated by matching tRNA pools than by mismatching tRNA pools, we compared the distribution of translation efficiencies for six different gene sets: (i) The whole transcriptome. (ii) The sets of the 200 most upregulated genes for a condition compared to all other dissimilar conditions in turn. Thus, for each condition we have established not just one gene set of upregulated genes, but several—one for each contrast. “Dissimilar” means that we compared healthy liver (and mouse E15.5) with either cancer cell line, but not the cancer cell lines between each other. To find the most upregulated genes, we performed differential expression analysis and picked the 200 genes with the lowest *p*-values, after discarding the lower three quartiles of genes by base mean expression. (iii) The gene sets created by the union of the significantly enriched GO terms in the same contrasts as for upregulated genes. (iv) The set of 408 housekeeping genes, taken from Zhu et al. [68]. For mouse, the same set of genes was used by translating the human gene names to mouse gene names. (v) The sets of ribosomal protein-coding genes, taken from Nakao et al. [69]. (vi) The set of proliferation driving genes derived from results published in Waldmann et al. [37], filtering for genes with a correlation-based cPI > 0 and *p* < 0.05. The same set was derived for mouse by translating the human gene names.

For each type of gene set, we performed the same analysis: For matching tRNA pools, we calculated the translational efficiency between the mRNA gene set and the tRNA pool for all pairwise combinations of replicates of the matching mRNA-seq and tRNA Pol III ChIP-seq data. For mismatching tRNA pools, we calculated the translational efficiency between the mRNA gene set and the tRNA pool for all pairwise combinations of replicates of RNA-seq data, and all replicates of dissimilar (see above) tRNA Pol III ChIP-seq data. Finally, we performed a one-tailed Mann–Whitney–Wilcoxon test between the distributions of matching and mismatching tRNA pools to determine whether we find any evidence that matching tRNA pools have higher translational efficiency.

Similar to Tuller et al. [7], translational efficiency of the start of the transcripts was analyzed by considering only the first 10 codons of each canonical transcript (instead of the whole transcript) and then calculating translational efficiency using the same method as before. To verify that a length of 10 codons yielded translational efficiencies that were representative of the ramp-up phase of translation, we tested different number of codons at the start of each transcript: from a minimum of 5 to a maximum of 25 in humans, and 26 in mouse (this corresponds to the length of the smallest canonical transcript in each dataset).

#### Codon usage PCA

To compare the variance in codon usage with the variance in translational efficiency, we performed a principal components analysis of the aggregate codon usage across GO terms. First, we calculated the aggregate codon usage for each GO term by summing over the codon frequencies of the canonical coding sequences of its genes. Next, we performed PCA on the matrix of codon usage against GO terms. We plotted the axis of largest variance (PC1) against the translational efficiency of each GO term gene set, calculated as described in the previous section. Next, for each condition we took the set of enriched GO terms and tested whether their translational efficiency was statistically higher than the translational efficiency of non-enriched GO terms using a one-tailed Mann–Whitney–Wilcoxon test. This was done for enriched GO terms in liver compared to either cancer cell line and mouse E15.5, in either cancer cell line compared to liver, and in mouse E15.5 compared to liver.

#### GC content

GO term GC content was calculated by concatenating the canonical coding sequences of all genes within a GO term gene set, and calculating their overall GC to ACGT frequency ratio. This was plotted against the PC1 of the codon usage PCA for each GO term.

## Supporting Information

S1 Fig Workflow of transcriptome-wide identification and analysis of protein-coding and tRNA genes. (A) RNA-seq analysis of protein-coding gene expression, differential expression analysis, gene set analysis and codon usage analysis. (B) ChIP-seq analysis of Pol III occupancy at tRNA gene loci and anticodon isoacceptor abundance.
(TIF)

S2 Fig Replicate correlations of RNA-seq in human liver cell types. Plots present the correlation of protein-coding gene expression level (log of raw counts in 1 000) between two biological replicates in human liver cell types (A–E). Spearman’s rank correlation coefficients (ρ) are reported in bottom right of each panel.
(TIF)

S3 Fig Replicate correlations of RNA-seq in mouse liver cell types. Plots present the correlation of protein-coding gene expression level (log of raw counts in 1 000) between two biological replicates in mouse liver cell types (A–I). Spearman’s rank correlation coefficients (ρ) are reported in bottom right of each panel.
(TIF)

S4 Fig Replicate correlations of Pol III ChIP-seq data in human liver cell types. Plots present the correlation of Pol III binding intensities to tRNA genes (log of raw counts in 1 000) between two biological replicates in human liver cell types (A–C). Spearman’s rank correlation coefficients (ρ) are reported in bottom right of each panel.
(TIF)

S5 Fig Replicate correlations of Pol III ChIP-seq data in human liver cell types. Plots present the correlation of Pol III binding intensities to tRNA genes (log of raw counts in 1 000) between two biological replicates in mouse liver cell types (A–D). Spearman’s rank correlation coefficients (ρ) are reported in bottom right of each panel.
(TIF)

S6 Fig Cell-type specific protein-coding and tRNA gene expression. Rows show cell-type specific expression of protein-coding genes in human (A–C) and mouse (D–F) as well as tRNA genes in human (G–I) and mouse (J–L). Left column: factorial map of the principal components (PC) analysis separates size factor normalized expression levels of global protein-coding genes (A and D) and tRNA genes (G and J). The proportion of variance explained by each principal component is indicated in parenthesis. Middle column: the 3-way (human) or 4-way (mouse) Venn diagram intersects the number of expressed protein-coding (B and E) and tRNA (H and K) genes. Areas are shaded according to cell-type. Right column: The intersection of the row/column for each cell type combination shows the proportion and number (in parenthesis) of differentially to all expressed protein-coding (C and F) and tRNA (I and L) genes. Top right triangle (yellow): up-regulated genes comparison from first, left to right and second, top to bottom. Bottom left triangle (blue): down-regulated genes comparison from first, top to bottom and second, left to right. Color gradient indicates proportional differences (0%: light, 100%: dark).
(TIF)

S7 Fig Mean gene expression levels of different gene groups. Violin plots represent the probability density of normalized expression levels (log10 transformed transcripts per million (TPM)) of protein-coding genes that are detectable in human (A) and mouse (B) liver (“all”), which are subdivided into genes that are (i) differentially expressed (“DE”) or in the top 200 fraction of those (“DEup”), (ii) associated with GO term with the most significant difference between any two conditions (“GO”) and (iii) encoding either house-keeping (“HK”), ribosomal (“RP”) or proliferation (“PP”) proteins. The median of the data is shown by a white dot, the interquartile range by a wide white line, and the first and third interquartile range by a thin white line. Gene numbers per group are shown in parenthesis.
(TIF)

S8 Fig Translational efficiencies in human and mouse liver cell-types. The data in this figure is the same as in Fig 3. Boxplots show transcriptomic mRNA codon usage and Pol III binding to tRNA isoacceptors correlations (translational efficiency) for all pairwise cell-type replicates for human (A) and mouse (B). Shown are the correlations of the codon pools for all genes (“all”), the 200 most highly differentially expressed (“DE”) protein-coding genes, condition-specific gene ontology (“GO”) term gene sets, house-keeping (“HK”), ribosomal (“RB”) or proliferation (“PP”) protein encoding genes. For each group, correlations were calculated with either the anticodon pool of the same condition (“M”) or any other condition (“MM”). For each data point, the identity of the cell type of its mRNA and tRNA are given in different colors and shapes, respectively. Asterisks above the bars indicate significant differences (significance codes of Bonferroni-corrected p-values: 0–0.001***, 0.001–0.01**, and 0.01–0.05*) between all gene groups by the one-tailed Mann–Whitney–Wilcoxon test.
(TIF)

S9 Fig tAI corrected translational efficiencies in human and mouse liver cell-types. The data in this plot is similar to that in Fig 3. Boxplots show translation efficiency, calculated as the tRNA adaptation index (tAI) using the transcriptomic mRNA codon usage and Pol III binding to tRNA isoacceptors for all pairwise cell-type replicates for human (A) and mouse (B) (Methods). Shown are tAIs of the codon pool for all genes (“all”), the 200 most highly differentially expressed (“DE”) protein-coding genes, condition-specific gene ontology (“GO”) term gene sets, house-keeping (“HK”), ribosomal (“RB”) or proliferation (“PP”) protein encoding genes with either the anticodon pool of the same condition (“M”) or any other condition (“MM”). For each data point, the identity of the cell type of its mRNA and tRNA are given in different colors and shapes, respectively. Asterisks above the bars indicate significant differences (significance codes of Bonferroni-corrected p-values: 0–0.001***, 0.001–0.01**, and 0.01–0.05*) between all gene groups by the one-tailed Mann–Whitney–Wilcoxon test.
(TIF)

S10 Fig Wobble base pairing corrected translational efficiencies in human and mouse liver cell-types. The data in this plot is similar to that in Fig 3. Boxplots show transcriptomic mRNA codon usage and Pol III binding to tRNA isoacceptors correlations (translational efficiency) for all pairwise cell-type replicates for human (A) and mouse (B). Correlations were corrected considering wobble-base pairing rules (Methods). Shown are correlations of the codon pool for all genes (“all”), the 200 most highly differentially expressed (“DE”) protein-coding genes, condition-specific gene ontology (“GO”) term gene sets, house-keeping (“HK”), ribosomal (“RB”) or proliferation (“PP”) protein encoding genes with either the anticodon pool of the same condition (“M”) or any other condition (“MM”). For each data point, the identity of the cell type of its mRNA and tRNA are given in different colors and shapes, respectively. Asterisks above the bars indicate significant differences (significance codes of Bonferroni-corrected p-values: 0–0.001***, 0.001–0.01**, and 0.01–0.05*) between all gene groups by the one-tailed Mann–Whitney–Wilcoxon test.
(TIF)

S11 Fig Translational efficiencies are lower within the first ten mRNA codons. The data in this plot is similar to that in Fig 3. Boxplots show translation efficiencies at the first 10 codons of each mRNA using the transcriptomic mRNA codon usage and Pol III binding to tRNA isoacceptors for all pairwise cell-type replicates for human (A) and mouse (B) (Methods). Shown are translational efficiencies of the codon pool for all genes (“all”), the 200 most highly differentially expressed (“DE”) protein-coding genes, condition-specific gene ontology (“GO”) term gene sets, house-keeping (“HK”), ribosomal (“RB”) or proliferation (“PP”) protein encoding genes with either the anticodon pool of the same condition (“M”) or any other condition (“MM”). Asterisks above the bars indicate significant differences (significance codes of Bonferroni-corrected p-values: 0–0.001***, 0.001–0.01**, and 0.01–0.05*) between all gene groups by the one-tailed Mann–Whitney–Wilcoxon test.
(TIF)

S12 Fig Translational efficiency does not correlate with cell-type-specific codon biases. Translation efficiency of each GO term for (A) all cell-types combined (black: adult liver, red: Huh-7 or Hepa1c1c7, yellow: HepG2 and Hepa1.6, blue: embryonic liver E15.5) or for (B) each cell-type alone is plotted against adult liver first principal component shown in (Fig 4A) (left: human cell types; right: mouse cell types). The proportion of variance explained by each PC is shown in parenthesis. The bar at the top corresponds to each GO term’s loading on PC1. The x-axes in (A) and (B) are identical. The y-axis shows the cell type-specific mean translation efficiency of each GO term. Black diamond symbols correspond to enriched GO terms (using gene set analysis of differentially expressed genes, empirical p < 0.001). Enrichment of adult liver is defined by contrast with cell lines and E15.5. Gray circles are non-enriched GO terms. The panel on the right hand side shows the density of the distributions of the translation efficiency of enriched (with a solid trace line) and non-enriched GO terms. The numbers at the bottom right of each plot correspond to the Bonferroni-corrected p-value of a one-sided Mann–Whitney–Wilcoxon test of the null hypothesis that no difference exists between the enriched and non-enriched GO term translational efficiency.
(TIF)

S1 Table GO terms of human genes. (TSV)

S2 Table Expression values of protein-coding genes in human liver cell types. (TSV)

S3 Table Expression values of protein-coding genes in mouse liver cell types. (TSV)

S4 Table Expression values of tRNA genes in human liver cell types. (TSV)

S5 Table Expression values of tRNA genes in mouse liver cell types. (TSV)

S6 Table Differentially expressed protein-coding genes in human liver cell types. (XLSX)

S7 Table Differentially expressed protein-coding genes in mouse liver cell types. (XLSX)

S8 Table Differentially expressed tRNA genes in human liver cell types. (XLSX)

S9 Table Differentially expressed tRNA genes in mouse liver cell types. (XLSX)

S10 Table Gene sets for human genes and p-values. (XLSX)

S11 Table Gene sets for mouse genes and p-values. (XLSX)

S12 Table GO analysis of differentially expressed protein-coding genes in human liver cell types. (XLSX)

S13 Table GO analysis of differentially expressed protein-coding genes in mouse liver cell types. (XLSX)
