# Supplementary figures and images for: Codon-Driven Translational Efficiency Is Stable across Diverse Mammalian Cell States
Source: PLoS Genet. 2016 May 11;12(5):e1006024. doi: 10.1371/journal.pgen.1006024 (PMC4864286; doi:10.1371/journal.pgen.1006024)

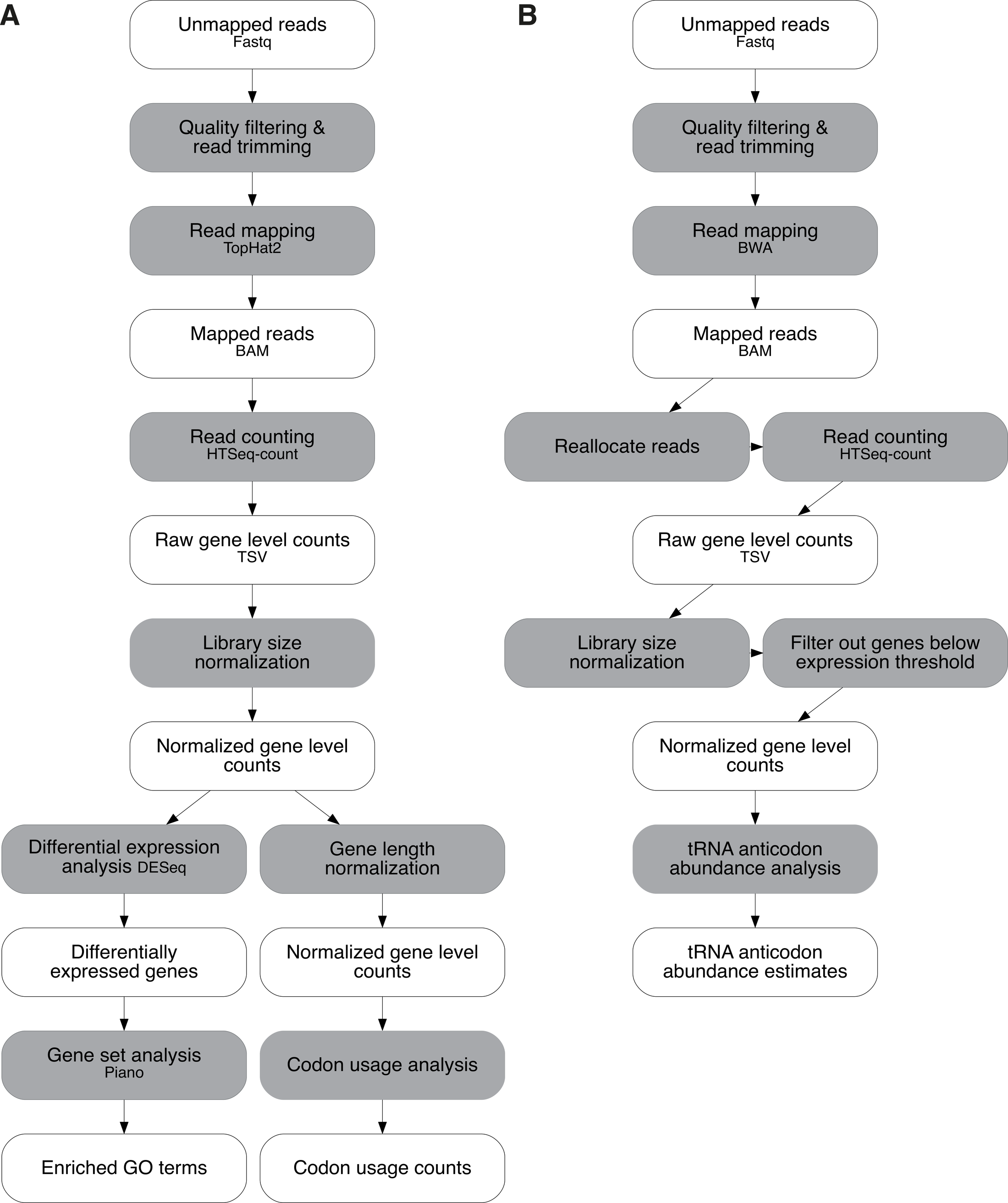

Supplement: S1 Fig — (A) RNA-seq analysis of protein-coding gene expression, differential expression analysis, gene set analysis and codon usage analysis. (B) ChIP-seq analysis of Pol III occupancy at tRNA gene loci and anticodon isoacceptor abundance. (TIF) [file pgen.1006024.s001.tif]

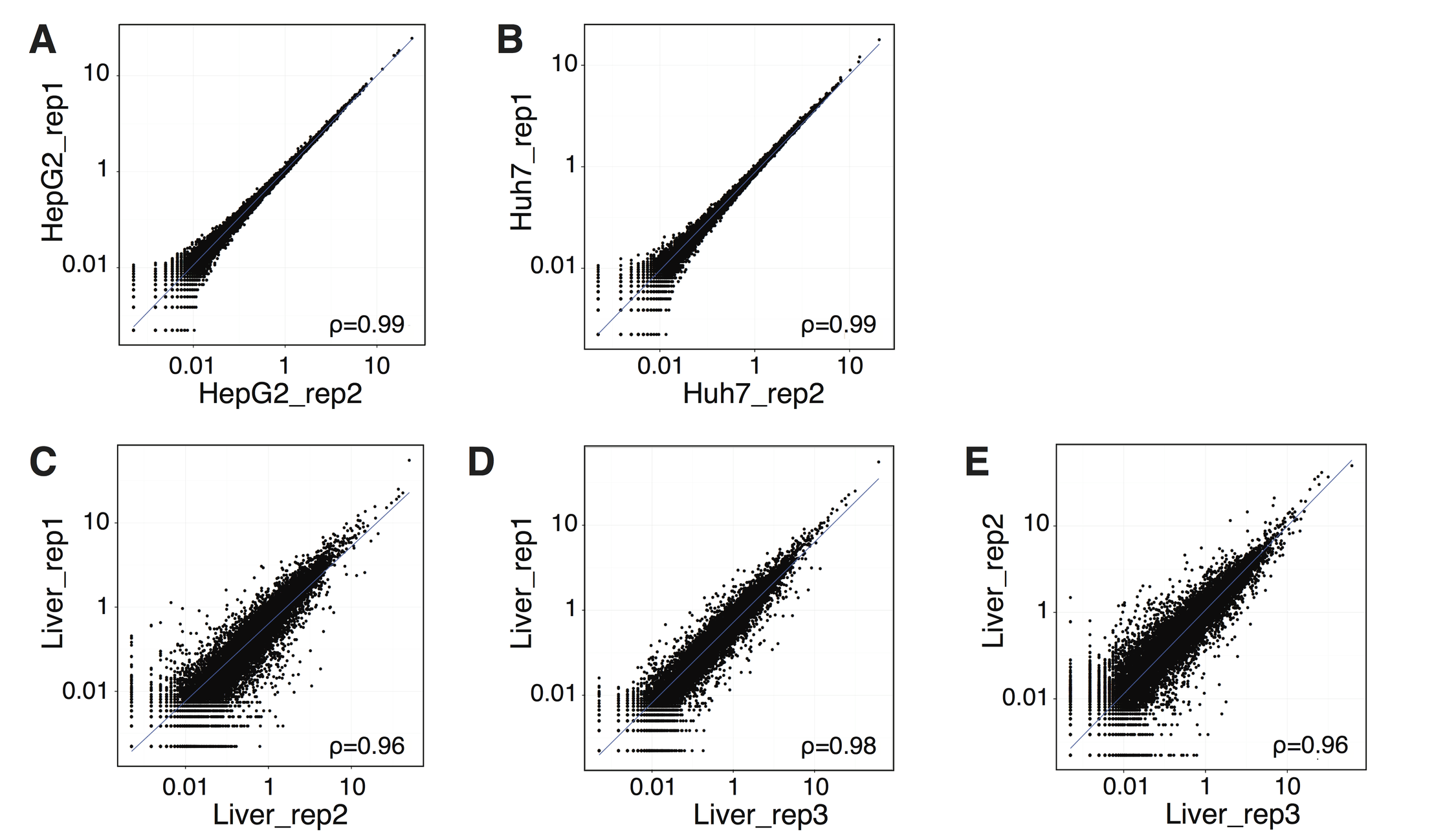

Supplement: S2 Fig — Plots present the correlation of protein-coding gene expression level (log of raw counts in 1 000) between two biological replicates in human liver cell types (A–E). Spearman’s rank correlation coefficients (ρ) are reported in bottom right of each panel. (TIF) [file pgen.1006024.s002.tif]

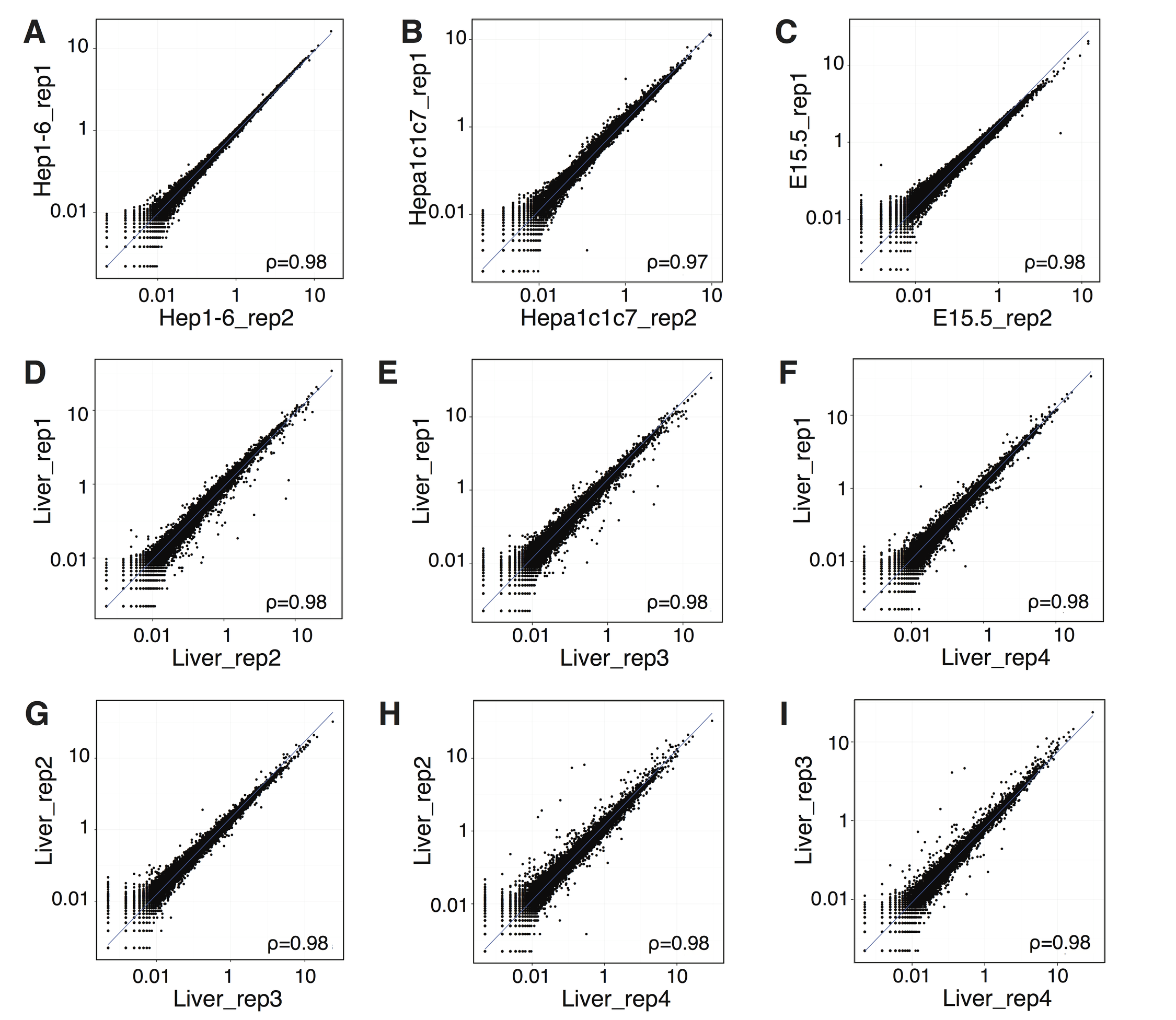

Supplement: S3 Fig — Plots present the correlation of protein-coding gene expression level (log of raw counts in 1 000) between two biological replicates in mouse liver cell types (A–I). Spearman’s rank correlation coefficients (ρ) are reported in bottom right of each panel. (TIF) [file pgen.1006024.s003.tif]

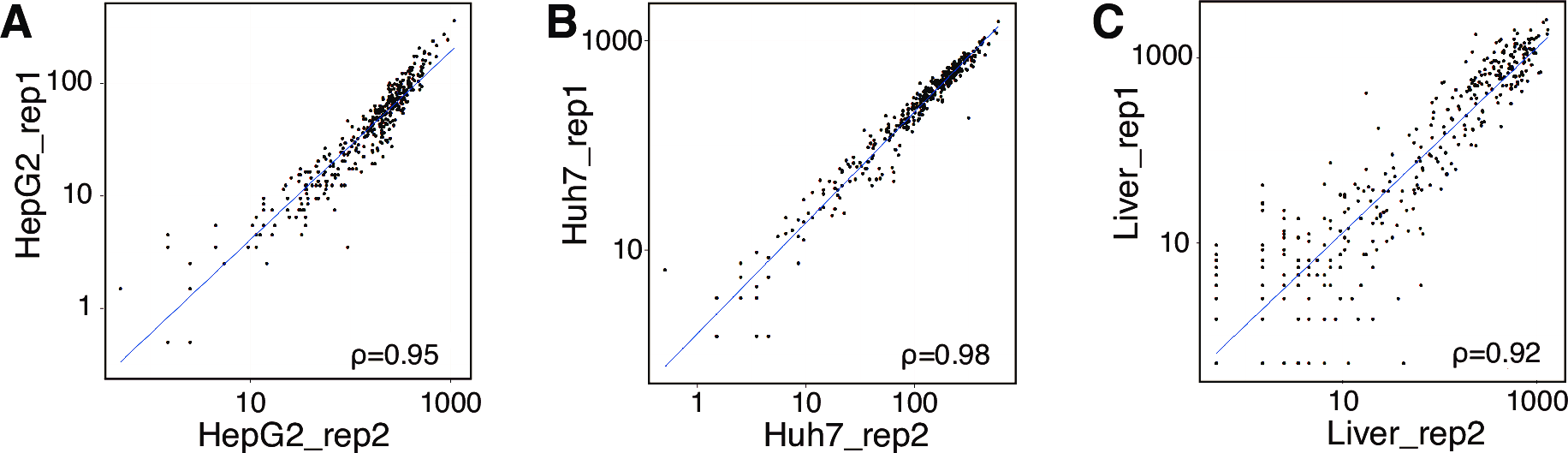

Supplement: S4 Fig — Plots present the correlation of Pol III binding intensities to tRNA genes (log of raw counts in 1 000) between two biological replicates in human liver cell types (A–C). Spearman’s rank correlation coefficients (ρ) are reported in bottom right of each panel. (TIF) [file pgen.1006024.s004.tif]

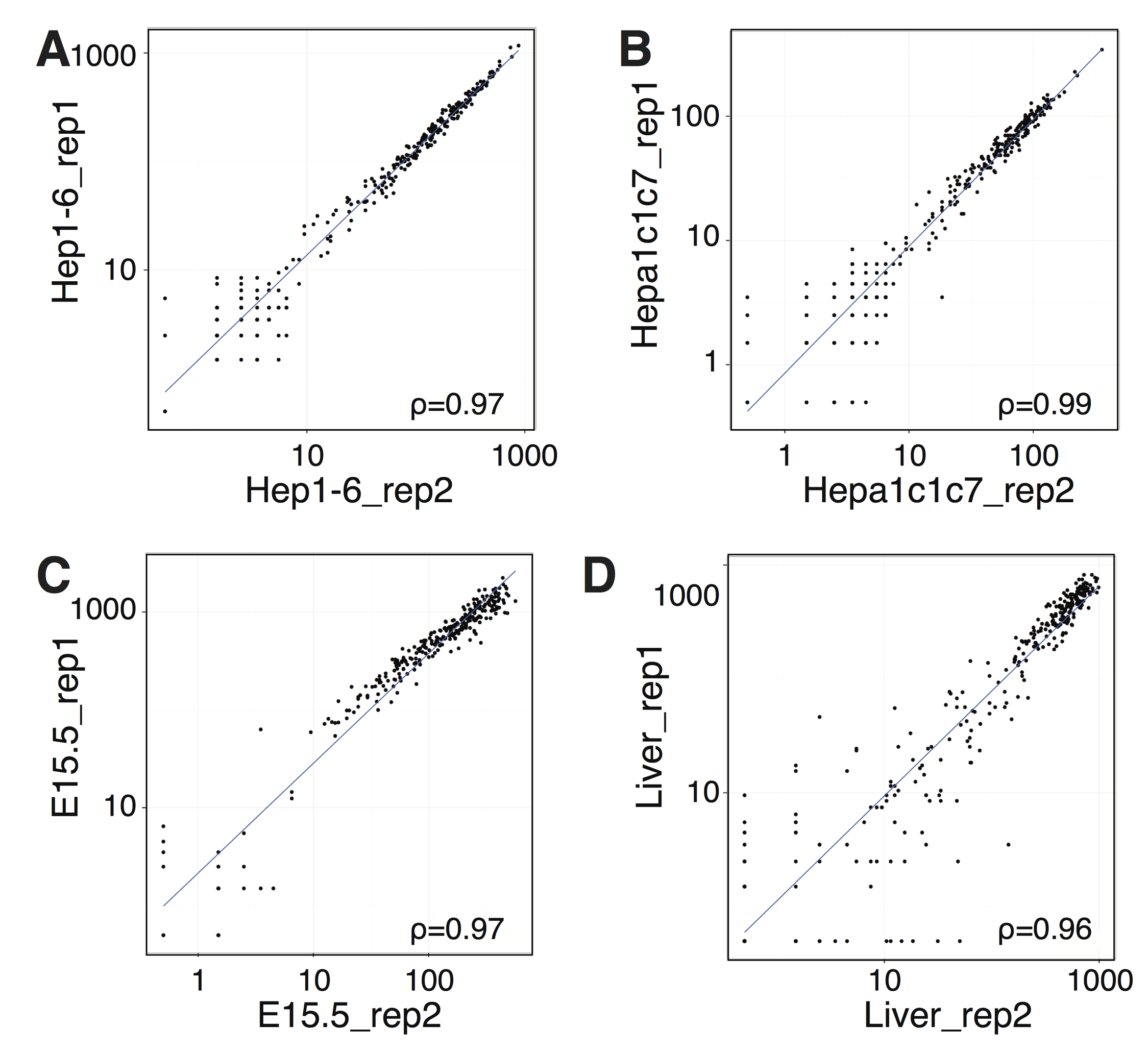

Supplement: S5 Fig — Plots present the correlation of Pol III binding intensities to tRNA genes (log of raw counts in 1 000) between two biological replicates in mouse liver cell types (A–D). Spearman’s rank correlation coefficients (ρ) are reported in bottom right of each panel. (TIF) [file pgen.1006024.s005.tif]

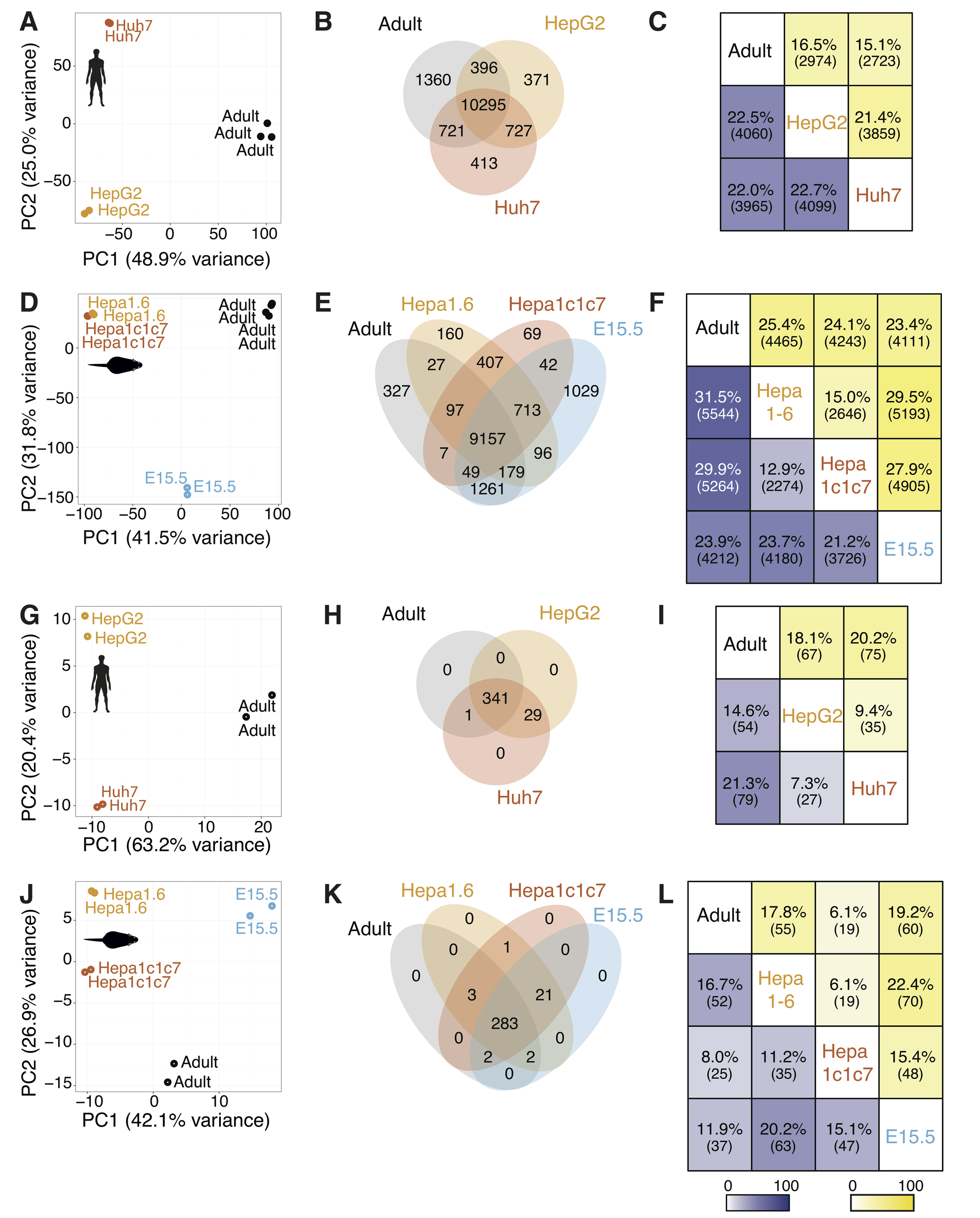

Supplement: S6 Fig — Rows show cell-type specific expression of protein-coding genes in human (A–C) and mouse (D–F) as well as tRNA genes in human (G–I) and mouse (J–L). Left column: factorial map of the principal components (PC) analysis separates size factor normalized expression levels of global protein-coding genes (A and D) and tRNA genes (G and J). The proportion of variance explained by each principal component is indicated in parenthesis. Middle column: the 3-way (human) or 4-way (mouse) Venn diagram intersects the number of expressed protein-coding (B and E) and tRNA (H and K) genes. Areas are shaded according to cell-type. Right column: The intersection of the row/column for each cell type combination shows the proportion and number (in parenthesis) of differentially to all expressed protein-coding (C and F) and tRNA (I and L) genes. Top right triangle (yellow): up-regulated genes comparison from first, left to right and second, top to bottom. Bottom left triangle (blue): down-regulated genes comparison from first, top to bottom and second, left to right. Color gradient indicates proportional differences (0%: light, 100%: dark). (TIF) [file pgen.1006024.s006.tif]

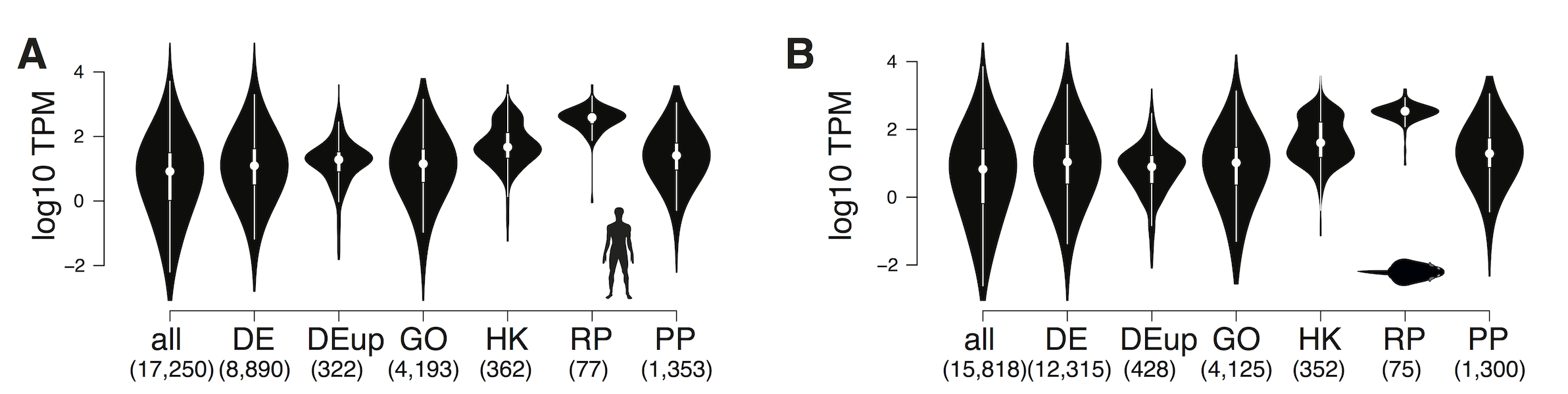

Supplement: S7 Fig — Violin plots represent the probability density of normalized expression levels (log10 transformed transcripts per million (TPM)) of protein-coding genes that are detectable in human (A) and mouse (B) liver (“all”), which are subdivided into genes that are (i) differentially expressed (“DE”) or in the top 200 fraction of those (“DEup”), (ii) associated with GO term with the most significant difference between any two conditions (“GO”) and (iii) encoding either house-keeping (“HK”), ribosomal (“RP”) or proliferation (“PP”) proteins. The median of the data is shown by a white dot, the interquartile range by a wide white line, and the first and third interquartile range by a thin white line. Gene numbers per group are shown in parenthesis. (TIF) [file pgen.1006024.s007.tif]

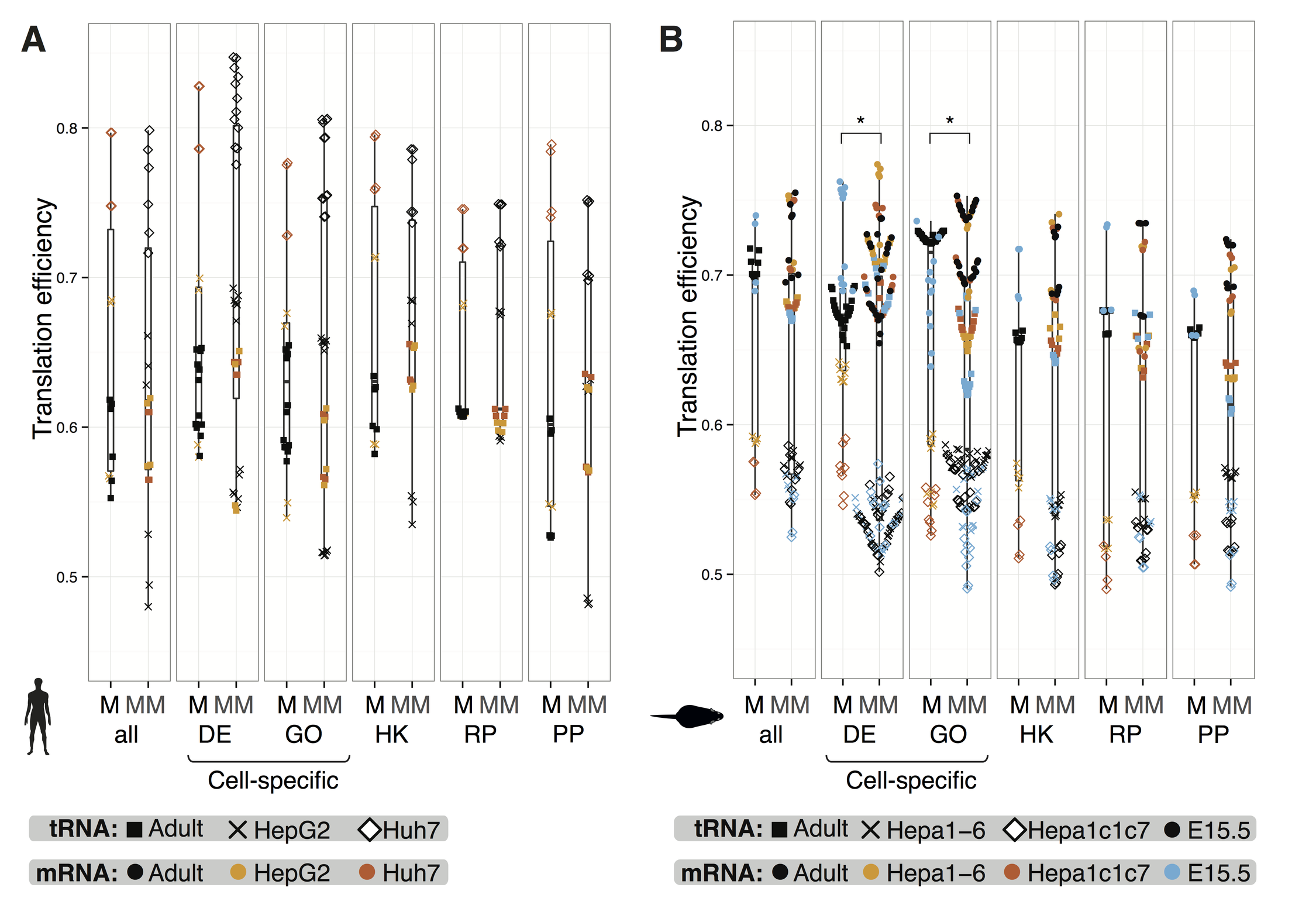

Supplement: S8 Fig — The data in this figure is the same as in Fig 3. Boxplots show transcriptomic mRNA codon usage and Pol III binding to tRNA isoacceptors correlations (translational efficiency) for all pairwise cell-type replicates for human (A) and mouse (B). Shown are the correlations of the codon pools for all genes (“all”), the 200 most highly differentially expressed (“DE”) protein-coding genes, condition-specific gene ontology (“GO”) term gene sets, house-keeping (“HK”), ribosomal (“RB”) or proliferation (“PP”) protein encoding genes. For each group, correlations were calculated with either the anticodon pool of the same condition (“M”) or any other condition (“MM”). For each data point, the identity of the cell type of its mRNA and tRNA are given in different colors and shapes, respectively. Asterisks above the bars indicate significant differences (significance codes of Bonferroni-corrected p-values: 0–0.001***, 0.001–0.01**, and 0.01–0.05*) between all gene groups by the one-tailed Mann–Whitney–Wilcoxon test. (TIF) [file pgen.1006024.s008.tif]

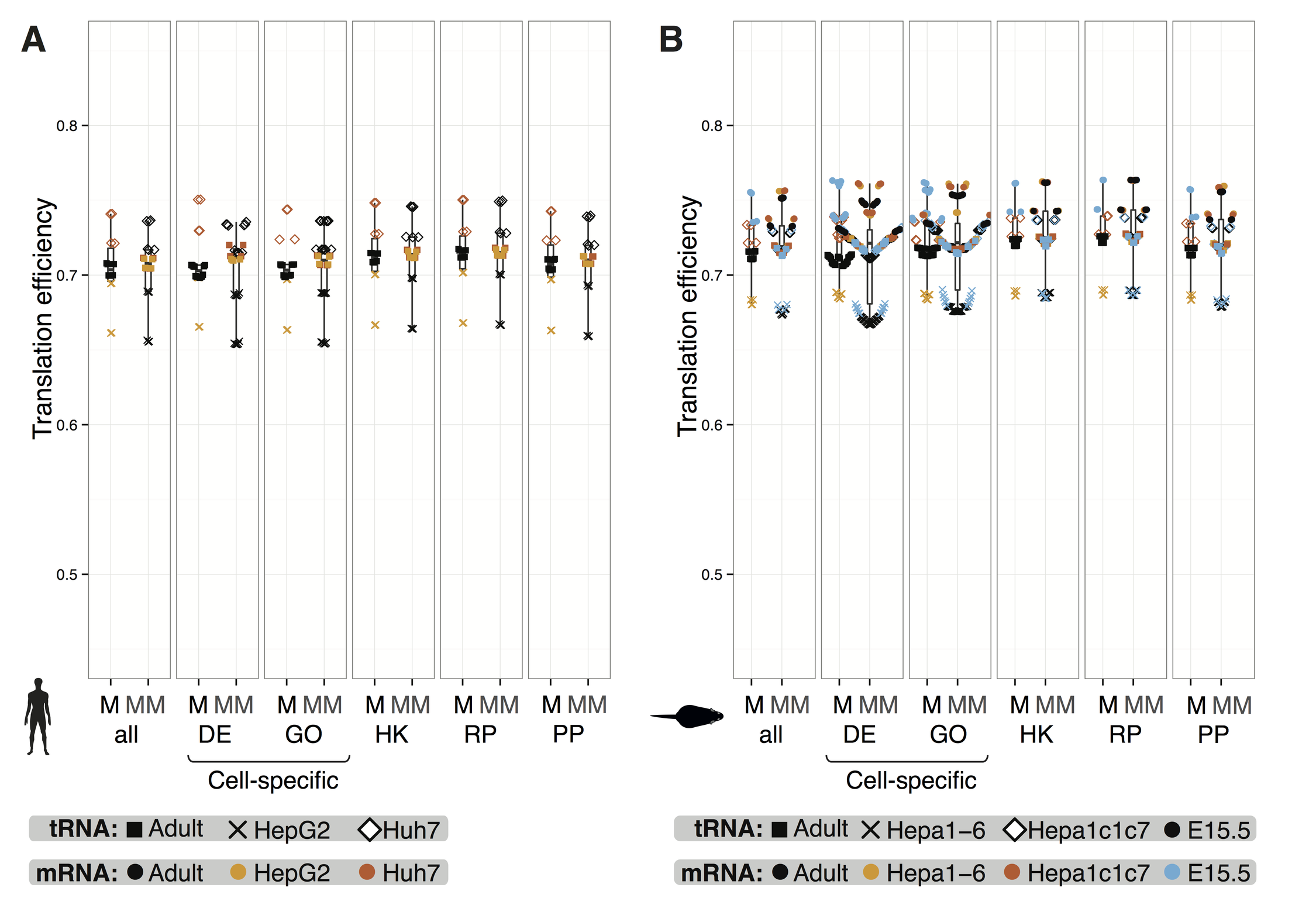

Supplement: S9 Fig — The data in this plot is similar to that in Fig 3. Boxplots show translation efficiency, calculated as the tRNA adaptation index (tAI) using the transcriptomic mRNA codon usage and Pol III binding to tRNA isoacceptors for all pairwise cell-type replicates for human (A) and mouse (B) (Methods). Shown are tAIs of the codon pool for all genes (“all”), the 200 most highly differentially expressed (“DE”) protein-coding genes, condition-specific gene ontology (“GO”) term gene sets, house-keeping (“HK”), ribosomal (“RB”) or proliferation (“PP”) protein encoding genes with either the anticodon pool of the same condition (“M”) or any other condition (“MM”). For each data point, the identity of the cell type of its mRNA and tRNA are given in different colors and shapes, respectively. Asterisks above the bars indicate significant differences (significance codes of Bonferroni-corrected p-values: 0–0.001***, 0.001–0.01**, and 0.01–0.05*) between all gene groups by the one-tailed Mann–Whitney–Wilcoxon test. (TIF) [file pgen.1006024.s009.tif]

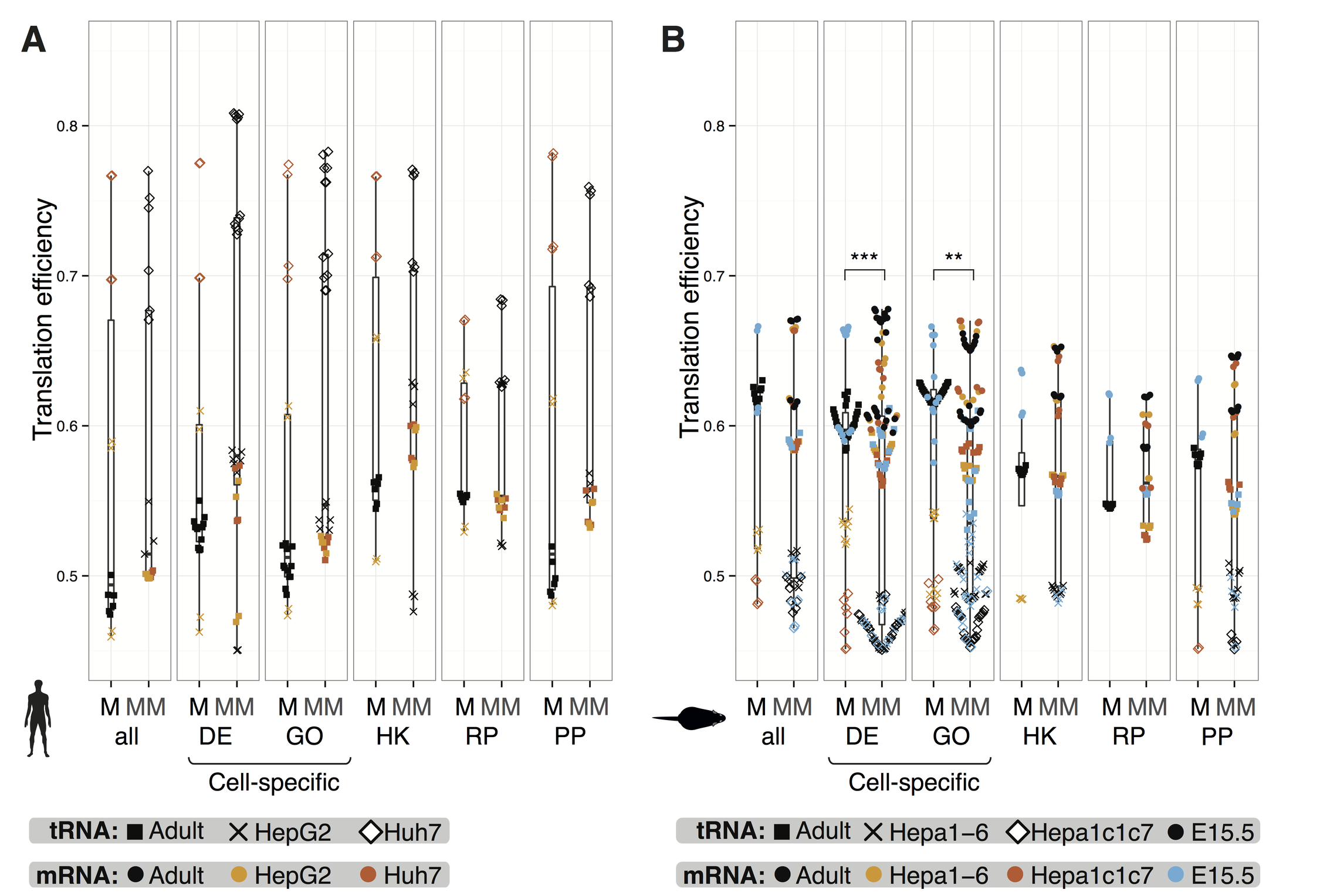

Supplement: S10 Fig — The data in this plot is similar to that in Fig 3. Boxplots show transcriptomic mRNA codon usage and Pol III binding to tRNA isoacceptors correlations (translational efficiency) for all pairwise cell-type replicates for human (A) and mouse (B). Correlations were corrected considering wobble-base pairing rules (Methods). Shown are correlations of the codon pool for all genes (“all”), the 200 most highly differentially expressed (“DE”) protein-coding genes, condition-specific gene ontology (“GO”) term gene sets, house-keeping (“HK”), ribosomal (“RB”) or proliferation (“PP”) protein encoding genes with either the anticodon pool of the same condition (“M”) or any other condition (“MM”). For each data point, the identity of the cell type of its mRNA and tRNA are given in different colors and shapes, respectively. Asterisks above the bars indicate significant differences (significance codes of Bonferroni-corrected p-values: 0–0.001***, 0.001–0.01**, and 0.01–0.05*) between all gene groups by the one-tailed Mann–Whitney–Wilcoxon test. (TIF) [file pgen.1006024.s010.tif]

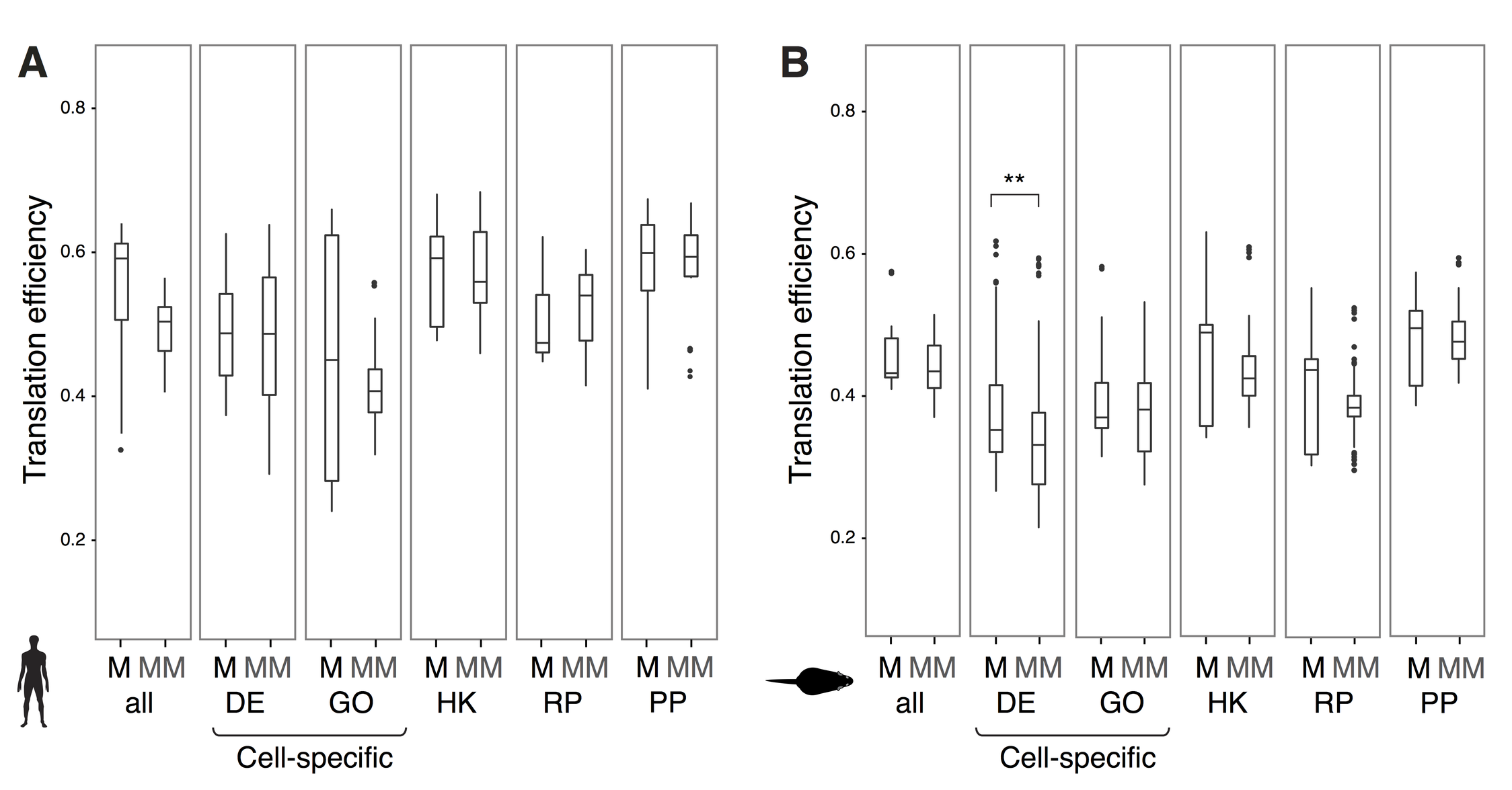

Supplement: S11 Fig — The data in this plot is similar to that in Fig 3. Boxplots show translation efficiencies at the first 10 codons of each mRNA using the transcriptomic mRNA codon usage and Pol III binding to tRNA isoacceptors for all pairwise cell-type replicates for human (A) and mouse (B) (Methods). Shown are translational efficiencies of the codon pool for all genes (“all”), the 200 most highly differentially expressed (“DE”) protein-coding genes, condition-specific gene ontology (“GO”) term gene sets, house-keeping (“HK”), ribosomal (“RB”) or proliferation (“PP”) protein encoding genes with either the anticodon pool of the same condition (“M”) or any other condition (“MM”). Asterisks above the bars indicate significant differences (significance codes of Bonferroni-corrected p-values: 0–0.001***, 0.001–0.01**, and 0.01–0.05*) between all gene groups by the one-tailed Mann–Whitney–Wilcoxon test. (TIF) [file pgen.1006024.s011.tif]

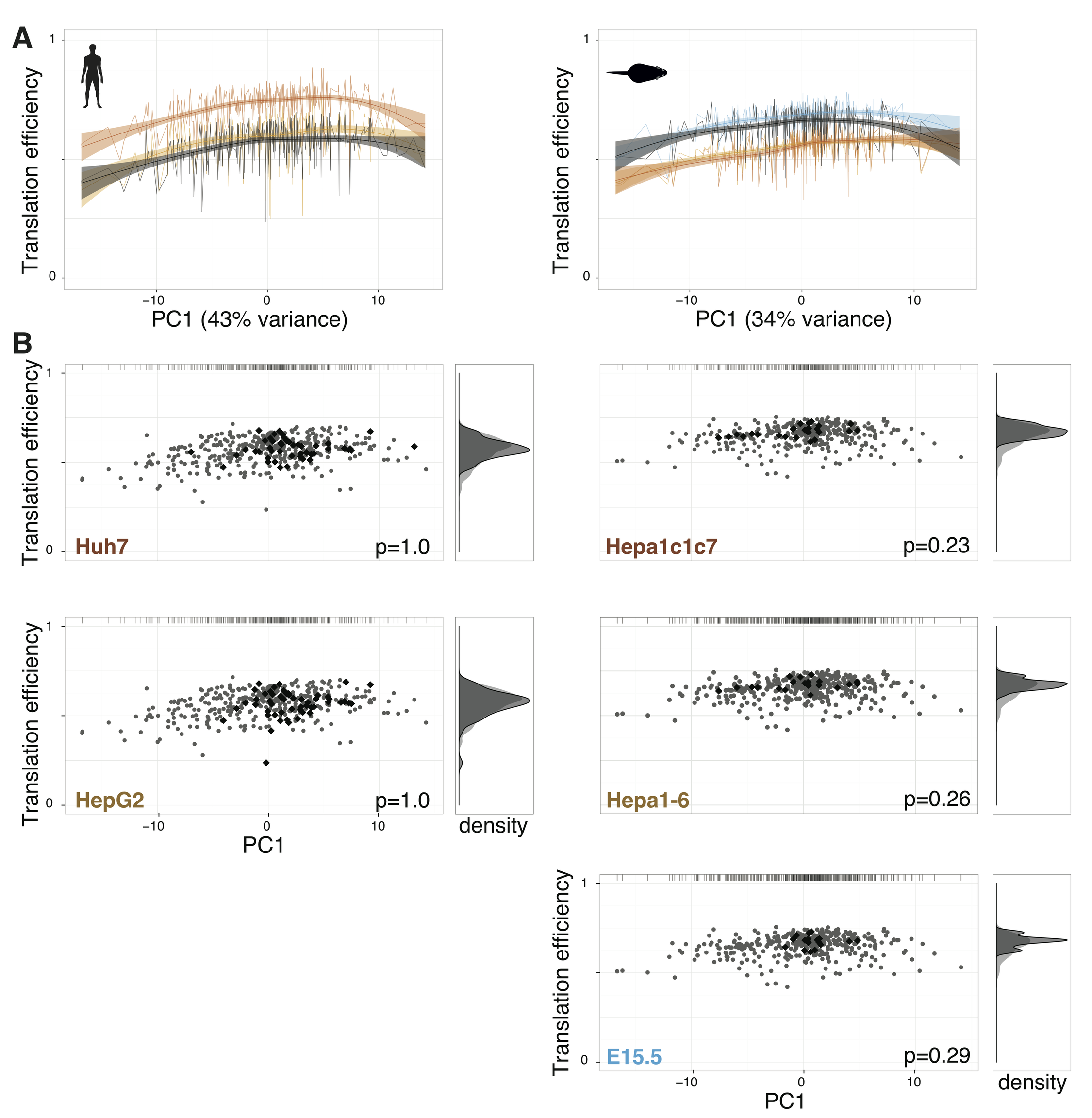

Supplement: S12 Fig — Translation efficiency of each GO term for (A) all cell-types combined (black: adult liver, red: Huh-7 or Hepa1c1c7, yellow: HepG2 and Hepa1.6, blue: embryonic liver E15.5) or for (B) each cell-type alone is plotted against adult liver first principal component shown in (Fig 4A) (left: human cell types; right: mouse cell types). The proportion of variance explained by each PC is shown in parenthesis. The bar at the top corresponds to each GO term’s loading on PC1. The x-axes in (A) and (B) are identical. The y-axis shows the cell type-specific mean translation efficiency of each GO term. Black diamond symbols correspond to enriched GO terms (using gene set analysis of differentially expressed genes, empirical p < 0.001). Enrichment of adult liver is defined by contrast with cell lines and E15.5. Gray circles are non-enriched GO terms. The panel on the right hand side shows the density of the distributions of the translation efficiency of enriched (with a solid trace line) and non-enriched GO terms. The numbers at the bottom right of each plot correspond to the Bonferroni-corrected p-value of a one-sided Mann–Whitney–Wilcoxon test of the null hypothesis that no difference exists between the enriched and non-enriched GO term translational efficiency. (TIF) [file pgen.1006024.s012.tif]
